# Mono- and Di-Pyrene [60]Fullerene and [70]Fullerene Derivatives as Potential Components for Photovoltaic Devices

**DOI:** 10.3390/molecules26061561

**Published:** 2021-03-12

**Authors:** Piotr Piotrowski, Wojciech Mech, Kamila Zarębska, Maciej Krajewski, Krzysztof P. Korona, Maria Kamińska, Magdalena Skompska, Andrzej Kaim

**Affiliations:** 1 Faculty of Chemistry, University of Warsaw, Pasteura 1, 02-093 Warsaw, Poland; ppiotrowski@chem.uw.edu.pl (P.P.); kzarebska@chem.uw.edu.pl (K.Z.); mskomps@chem.uw.edu.pl (M.S.); 2 Faculty of Physics, University of Warsaw, Pasteura 5, 02-093 Warsaw, Poland; Wojciech.Mech@fuw.edu.pl (W.M.); Maciej.Krajewski@fuw.edu.pl (M.K.); Krzysztof.Korona@fuw.edu.pl (K.P.K.); Maria.Kaminska@fuw.edu.pl (M.K.)

**Keywords:** fullerene, pyrene, acceptor, photovoltaics

## Abstract

In the present work, we report the successful synthesis and characterization of six (two new) fullerene mono- and di-pyrene derivatives based on C_60_ and C_70_ fullerenes. The synthesized compounds were characterized by spectral methods (ESI-MS, ^1^H-NMR, ^13^C-NMR, UV-Vis, FT-IR, photoluminescence and photocurrent spectroscopy). The energy of HOMO and LUMO levels and the band gaps were determined from cyclic voltammetry and compared with the theoretical values calculated according to the DFT/B3LYP/6-31G(d) and DFT/PBE/6-311G(d,p) approach for fully optimized molecular structures at the DFT/B3LYP/6-31G(d) level. Efficiency of solar cells made of PTB7: C_60_ and C_70_ fullerene pyrene derivatives were analyzed based on the determined energy levels of the HOMO and LUMO orbitals of the derivatives as well as the extensive spectral results of fullerene derivatives and their mixtures with PTB7. As a result, we found that the electronic and spectral properties, on which the efficiency of a photovoltaic cell is believed to depend, slightly changes with the number and type of pyrene substituents on the fullerene core. The efficiency of constructed solar cells largely depends on the homogeneity of the photovoltaic layer, which, in turn, is a derivative of the solubility of fullerene derivatives in the solvent used to apply these layers by spincoating.

## 1. Introduction

In the last few years, a variety of fullerene derivatives have been intensively studied, especially for the needs of electronic materials [1]. For this purpose, a number of fullerene derivatives have been achieved by replacing the aromatic and/or aliphatic substituent in the most popular and most commonly used soluble C_60_ derivative, [6,6]-phenyl-C61-butyric acid methyl ester (PC60BM) [2]. Among them, the fullerene buckyballs with attached polycyclic aromatic substituents aroused a great interest because the extended π-conjugated groups are considered to lend the material unusual and desired physicochemical properties.

In particular, the pyrene derivatives with their planar aromatic hydrocarbon fused rings were found especially attractive candidates for numerous applications, such as acceptors in bulk heterojunction solar cells [3], the additives in bulk-heterojunction solar cells containing C_60_ or C_70_ acceptors for preventing the aggregation of fullerenes [4], photosensitizers for the generation of the excited singlet oxygen state in photo stimulated processes with applications in energy transport systems [5], and white light emitters [6]. Furthermore, the pyrene-[60]fullerene dyads were shown to operate as UV light-harvesting materials where pyrenes acted as antennas [7].

The beneficial properties of pyrene-fullerene systems certainly origin in part from pyrene components that exhibits a number of structural and electronic features promising for construction of dye sensitized solar cells [8], blue light emitters [9,10], component of acceptor-donor-acceptor materials for efficient photovoltaic semiconductors [11], dispersant of neat fullerenes [12] and chemical sensing agent for fullerenes with fluorescence spectroscopy due to supramolecular complexes formation with C_60_ and C_70_ fullerene [13].

Despite a few reports on attempts to use pyrene-fullerene derivatives for organic solar cells (OSCs), the impact of the pyrene component on the power conversion efficiency is unclear. On one hand, it was found that due to the formation of pyrene-fullerene complexes, the polymer solar cells based on poly(3-hexylthiophene) as photoactive material and fullerene:pyrene acceptor showed efficiency of 1.54% and 2.50% for C_60_ and C_70_, respectively [4]. Similarly, in another study it was shown that fullerene C_60_ with an aryl substituent used as an electron acceptor was successfully used in photovoltaic devices, with the energy conversion efficiency strongly dependent on the type of the aryl substituent, achieving a relatively low efficiency of 1.95% for the acid methyl ester derivative [6,6]-pyrenyl-C61-butyric versus 3.80, 4.09 and 1.14% for phenyl, fullerene derivatives substituted with naphthalenyl and anthracenyl, respectively [3]. Very recently, pyrene-imidazole derivative (PyPI), which can form an efficient π-π stacking in solid, has been used to facilitate a charge transfer and suppress the fullerene aggregation. As a result, a ternary OSC based on polymeric PTB7-Th donor:PyPI:PC70BM achieved the power conversion efficiency (PCE) as high as 10.36% [14].

On the other hand, it has been postulated that the presence of the pyrene derivatives in the photovoltaic blends made of conjugated polymer PTB7 as the electron donor and neat fullerenes C_60_ and C_70_ as the electron acceptors adversely affects the efficiency of photovoltaic cells because of electron trapping by the pyrene substituent. This leads to unbalanced charge transport in the photovoltaic material, i.e., undisturbed transport of the holes and hindered electron hopping by the intercalation of fullerenes with pyrene moieties [12]. Based on other studies, it has also been shown that for fullerenes and metallofullerenes, tight and ordered packing is beneficial for intermolecular charge transport and energy transfer [15].

**Figure 1:**
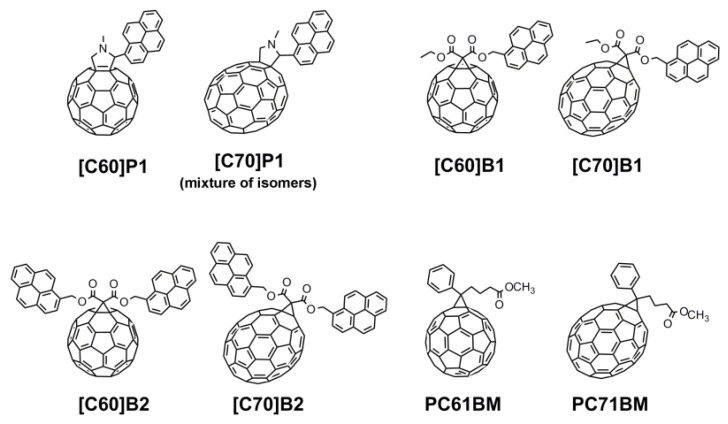
Structures of investigated C_60_ and C_70_ fullerene–pyrene derivatives (in the case of [C70]P1 only structure of one of the possible isomers is shown; in performed experiments mixture of all synthesized isomers was used).

Therefore, the aim of this paper was to clarify the influence of the structure of fullerene-pyrene derivatives such as the number of pyrene substituents, the type of link between pyrene moieties and the fullerene bowl, as well as the size of the fullerene itself on the electronic and photochemical properties of the conjugated system. In order to achieve this goal, three C_60_ ([C60]P1, [C60]B1, [C60]B2) and corresponding three C_70_ fullerene pyrene derivatives ([C70]P1, [C70]B1, [C70]B2) were synthesized using Prato [16] and Bingel [17] procedures (Figure 1). The experimentally obtained electronic data (HOMO and LUMO orbital energies and the band gap, E_g_) for all compounds were correlated with quantum-mechanical results obtained by DFT calculations with the use of two density functional methods PBE and B3LYP. The PBE functional approach was chosen because it has been proven to reproduce reliable data for fullerene systems [18]. For comparison, the B3LYP method was also used as one of the most widely applied computational method for comparison with experimental results [19] anticipating, however, that according to numerous literature data, the PBE will give results more in line with the experiment.

## 2. Results and Discussion

### 2.1. HOMO and LUMO Energies

#### 2.1.1. Determination of HOMO and LUMO Levels from CV Measurements

**Figure 2:**
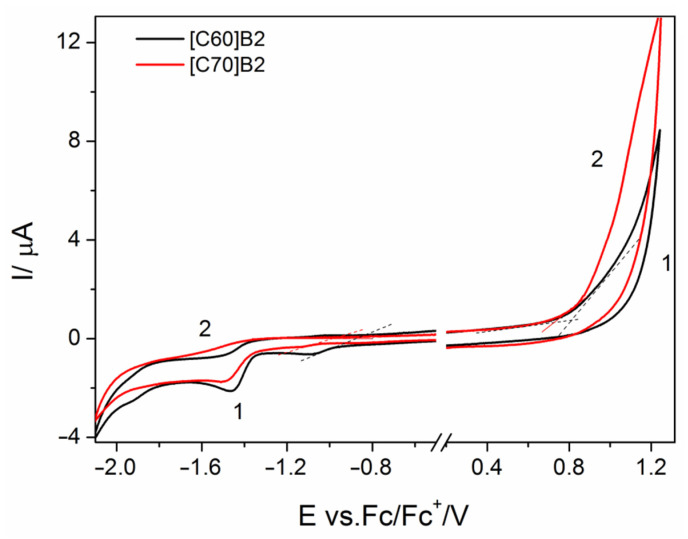
Cyclic voltammograms on Pt electrode in the solutions of [C60]B2 (1) and [C70]B2 (2) in CH_2_Cl_2_ containing 0.1 M TBAPF_6_ supporting electrolyte.

The onset potentials of oxidation and reduction of the fullerene derivatives ($E_{onset}^{ox}E_{onset}^{red}$, respectively), at which the initial injection of holes to HOMO level or electrons to LUMO level occurs, were determined from the onsets of anodic and cathodic currents in the cyclic voltammograms recorded at the scan rate of 100 mV s^−1^. The exemplary cyclic voltammograms recorded in the solutions of [C60]B2 and [C70]B2 are presented in Figure 2 (remaining cyclic voltammograms can be found in Supplementary Materials, Figures S1 and S2).

It is worth noting that the oxidation and reduction processes are irreversible. In order to prevent the influence of the reaction products on the course of the cyclic voltammograms, the measurements were performed separately in the positive and negative potential ranges, starting from the open circuit potential, and the electrode was cleaned before each measurement. The onset potentials of oxidation and reduction processes were referred to the potential of standard hydrogen electrode (SHE), as described above, and converted into the absolute scale (in eV), assuming that 0 V vs. SHE corresponds to −4.43 eV [20]. Thus, the *E_LUMO_* and *E_HOMO_* levels were determined from the equations:

(1) $$E_{LUMO}=-\left(eE_{onset}^{red}+4.43\mathrm{eV}\right)$$

(2) $$E_{HOMO}=-\left(eE_{onset}^{ox}+4.43\mathrm{eV}\right)$$

The values of *HOMO* and *LUMO* levels determined from the cyclic voltammograms for the series of C_60_ and C_70_ fullerene derivatives are listed in Table 1.

As is visible, the position of LUMO level is not strongly influenced by the type of linkage between pyrene group and fullerene. Some decrease is observed after attachment of the second pyrene unit. A stronger dependence is observed in case of HOMO level. A higher value was found for derivative with a linkage via nitrogen then via ester bond. Attachment of the second pyrene unit leads to the further lowering of HOMO level. The trend of the changes is similar for both fullerene derivatives but the differences are higher for C_60_ than for C_70_ derivatives.

#### 2.1.2. Theoretical HOMO and LUMO Frontier Orbitals by PBE and BLYP Functional Calculations

**Figure 3:**
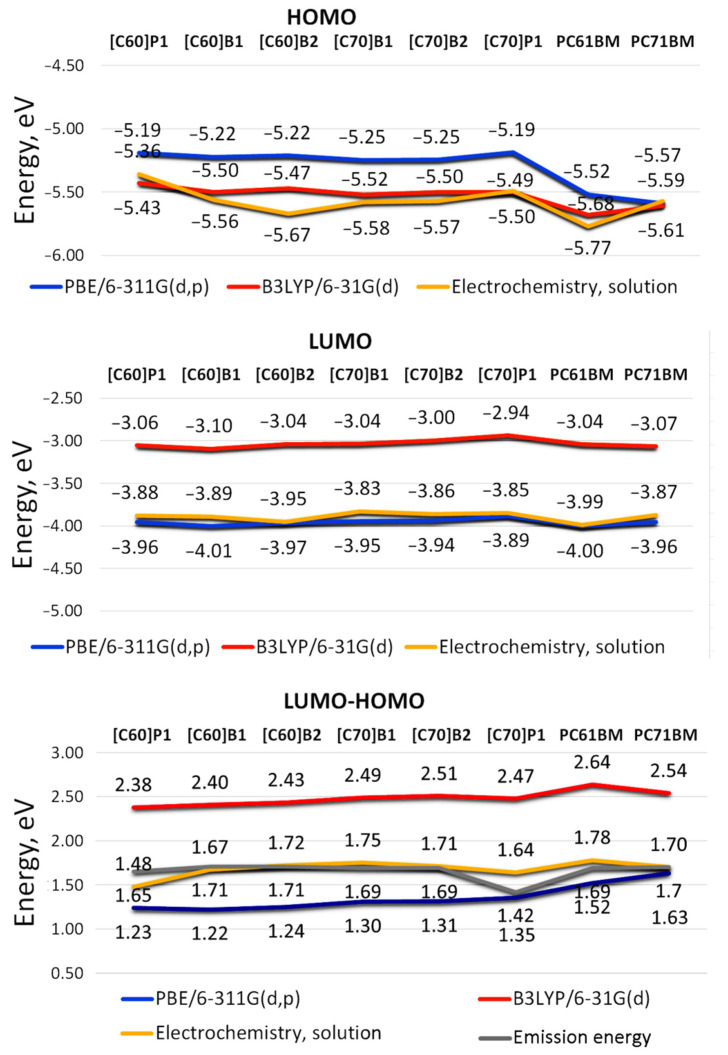
Calculated by DFT/B3LYP/6-31G(d) and DFT/PBE/6-311G(d,p) method and experimentally measured by cyclic voltammetry and luminescence spectroscopy HOMO, LUMO and LUMO-HOMO energies for investigated pyrene C_60_ and C_70_ fullerene derivatives. The HOMO-LUMO energy gaps of PC60BM calculated with B3LYP and PBE method are similar to the results in the literature [22].

In order to investigate a correlation between the structure of pyrene C_60_/C_70_ fullerene derivatives and theoretical frontier orbitals HOMO and LUMO, two levels of theory were used; the generalized gradient approximation (GGA) functional PBE and the B3LYP exchange-correlation functional with 6-311G(d,p) and 6-31G(d) basis set, respectively. The obtained theoretical results were then compared with the experimental results from cyclic voltammetry (Figure 2). Note that in the case of compound [C70]P1, Figure 3 shows the theoretical weighted average result for the mixture of four isomers that were formed during the synthesis of this compound similarly as reported in the literature (see Supplementary Materials, Table S1 and Figure S3) [21].

It can be seen that the HOMO energy levels obtained with the PBE and B3LYP functional are generally more similar to each other (Δ = |HOMO_PBE_ − HOMO_B3LYP_| = 0.02 − 0.30 eV) than those of LUMO orbitals (Δ = |LUMO_PBE_ − LUMO_B3LYP_| = 0.89 − 0.96 eV). Interestingly, theoretical HOMO results for two benchmark acceptor compounds calculated here for comparison; namely, PC60BM and PC70BM, often used in organic solar cells as an electron acceptor [23,24,25] show comparatively lower energies than those of remaining C_60_/C_70_ compounds containing pyrene substituent(s). At the same time, the corresponding LUMO energy values of the reference compounds are for the most part higher than those of the studied pyrene [60]fullerene and [70]fullerene derivatives. Qualitatively, very similar dependencies were observed using DFT method with B3LYP exchange correlation (XC) and PBE functional in combination with basis set 6-31G(d) of another system of conjugated double bonds (coumarin) and its derivatives. The HOMO and LUMO energies estimated according to both theoretical approaches remained in similar relationships as in this study [26].

The different performance of the PBE and B3LYP density functional is probably related to the Hartree–Fock (HF) exchange (eX%) contribution included. It is generally believed that the pure or hybrid functionals with low or no eX% such as PBE with eX% = 0 and B3LYP with eX% = 20 [27], produce significantly overestimated HOMO energies and underestimated LUMO energies due to overdelocalization (or overlocalization) of the HOMO and LUMO orbitals [28]. The number of carbon atoms making up the π bond system in the tested compounds does not seem to have a significant impact on the energy of the HOMO and LUMO orbitals. Single-walled C_20_nH_20_ nanotubes with n = 2–6 tested using the same functionalities showed for C_60_H_20_ to C_100_H_20_ systems a similar independence of frontier orbital energies from the number of carbon atoms [29].

An obvious consequence of the different frontier orbital HOMO and LUMO energies obtained with the PBE and B3LYP functional is the difference in LUMO-HOMO gaps for both theoretical approaches. Comparison of the theoretical energy levels for the investigated C_60_/C_70_ fullerene derivatives shows that LUMO-HOMO energy gaps obtained by B3LYP method are higher by value 0.91–1.20 eV when compared with those calculated by PBE method, however the smallest differences in orbital energy gaps from both methods were noted for PC60BM and PC70BM, thus compounds devoid of pyrene substituent(s). The obtained HOMO-LUMO results are consistent with the published data for PCBM-like C_60_ derivatives for which the HOMO-LUMO gaps were calculated in the range of 1.52–1.58 eV and 2.54–2.60 eV for the PBE [30] and B3LYP [31] functional, respectively.

#### 2.1.3. Theoretical vs. Experimental LUMO and HOMO Energies

Although it is believed that quantum mechanical calculations, and especially those that use the modern DFT method of density functionals, are a good tool to rationalize the experimental data and to predict electronic properties of designed organic molecules, there are many reasonable objections to the use of hybrid functionals for this purpose [32]. Nevertheless, the global-hybrid exchange–correlation density functional such as B3LYP functional is still the most popular functional in many areas of organic quantum chemistry, despite its known shortcomings [33]. Thus, attempts to apply this theoretical approach to the design of new organic derivatives are still on the way [34]. However, it was proved that the HOMO and LUMO energies, HOMO-LUMO gap, and bond length for C_60_ obtained from PBE functional agree far better with experimental results than those from B3LYP functional. It has been also suggested that PBE functional yields more reliable results for PC60BM-like fullerene derivatives while the most commonly used B3LYP functional performs rather poorly [32]. In the case of non-fullerene extended systems of conjugated double bonds C=C, this correlation may be inverse [8].

However, in the case of the studied fullerene pyrene derivatives, despite the unfavorable opinion, the DFT/B3LYP/6-31G(d) approach reflects the experimental HOMO orbital better than the DFT/PBE/6-311G (d, p) method (Figure 2). Interestingly, for the [C60]B2 molecule, HOMO sphere is evenly delocalized on both pyrene moieties, probably due to symmetry of the molecule. For the molecule [C70]B2, the HOMO orbital is predominantly located on one of the pyrene moieties, the one whose distance to the fullerene sphere is bigger. 

However, in the case of LUMO orbitals, the energies calculated by means of B3LYP are higher by a value of 0.7–1.2 eV than those measured experimentally, while the results of PBE correspond very well with the results of voltammetric measurements. Thus, the LUMO energy of the fullerene materials containing aromatic ring substituents can be easily accessed by simple DFT/PBE/6-311G(d,p) calculations. This seems important as the acceptor LUMO orbital level is critical to the open-circuit voltage (*V_OC_*) [35]:

(3) $$V_{oc}=\frac{1}{e}\left(E_{LUMO}^{Fullerene}-E_{HOMO}^{Polymer}-\Delta \right)+\frac{kT}{e}\ln\left(\frac{n_{e}n_{h}}{N_{c}^{2}}\right)$$

where *n_e_* and *n_h_* are the electron and hole densities in fullerene and polymer domains, respectively, *N_c_* is the density of conduction states at the band edge of the polymer and fullerene approximately both approximately equal. Δ stands for the exciton binding energy determining the ultimate device efficiency [36]. It should be noted however, that the pyrene C_60_ and C_70_ fullerene derivatives examined in this study do not differ significantly in the value of LUMO orbitals potentials. From the point of view of the activity of the tested materials in solar cells, this means that the presence of various substituents with pyrene rings in C_60_ and C_70_ fullerene derivatives does not significantly affect their $\left(E_{LUMO}^{Fullerene}-E_{HOMO}^{Polymer}\right)$ gap, thus *V_OC_* of devices made of them. Therefore, as in the case of voltammetric results, it can be concluded that the slight differences in the theoretical differences in the energy of the HOMO and LUMO orbitals associated with differently modified C_60_ and C_70_ fullerenes should not have a significant impact on the efficiency of the cells made of them. The spatial distributions of the HOMO/LUMO orbitals of the studied molecules seem to confirm this assumption.

**Figure 4:**
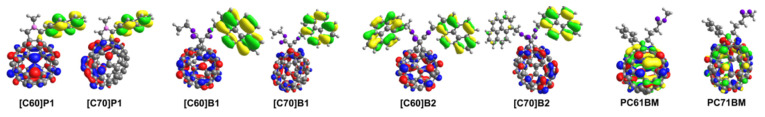
Models of HOMO (green and yellow) and LUMO (red and blue) level orbitals for optimized fullerene derivatives. Yellow and red lobes correspond to positive whereas green and blue lobes to negative isosurface values.

The modeled frontier HOMO/LUMO orbitals of the investigated molecules are presented in Figure 4. Analysis of the frontier orbitals distribution of the pyrene containing compounds according to B3LYP/6-31G(d) level of theory shows that the frontier HOMOs are only located on pyrene moieties, while the LUMO orbital responsible for the electron accepting properties is only located on the fullerene bowl, as in the case of C_60_ and C_70_ fullerene and other fullerene acceptors [21].

The only exception are derivatives of PC60BM and PC70BM, for which both HOMO and LUMO orbitals are located on the fullerene sphere. Although the LUMO orbitals of pyrene derivatives are always observed on the fullerene ball, due to the asymmetric structure of C_70_ derivatives only part of the volume of the fullerene sphere is the distribution site of this orbital (Figure 4). The reason for this uneven distribution of the LUMO orbital on the fullerene cup is the interaction of the π-π of the proximal pyrene substituent with the fullerene core [12]. However, it seems that this does not significantly affect the LUMO energy level of C_70_ pyrene derivatives (see below).

### 2.2. Properties of Solar Cells

#### 2.2.1. Current-Voltage Characteristics of Solar Cells

**Figure 5:**
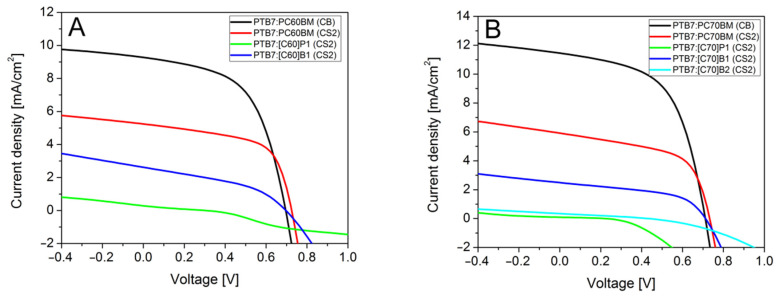
Current-voltage characteristics of solar cells with fullerene derivatives: (**A**) C_60_, (**B**) C_70_.

Current-voltage measurements (Figure 5) showed that the highest power conversion efficiencies were obtained for the reference solar cells containing PTB7:PC60BM or PTB7:PC70BM active layer.

As can be found in Table 2, the maximum efficiencies were 3.59% and 4.59% for PC60BM and PC70BM derivatives, respectively. For both cells, we could see almost the same fill factor and internal resistances which indicate that quality of both cells was similar and differences comes only form different fullerenes derivatives. For PTB7:PC60BM and PTB7:PC70BM dissolved in carbon disulfide solvent, we observed efficiencies of 2.30% and 2.49%, respectively. The PCE of solar cells with active layers obtained from CS_2_ solution was lower (compared to layers from chlorobenzene solutions) since the layers were thinner, due to less concentrated solution. Thinner layers absorbed fewer photons, so the photogenerated current was lower but, at the same time, showed less series resistance, which generally improves PCE. These cells also showed high *V_OC_* and FF. Analysis of I-V curves showed that among the new fullerene derivatives only [C60]B1 and [C70]B1 worked efficiently in solar cells. Their maximum efficiencies were 0.75% and 0.90%, for [C60]B1 and [C70]B1, respectively. The efficiency was diminished mainly by lower J_sc_ (2 times) and by increased serial resistance. Probably, the conductivity of [C60]B1 and [C70]B1 fullerene derivatives is lower compared to PCBM. On the other hand, high FF testifies to suitable polymer-fullerene phase separation. Solar cells with fullerene derivative [C70]B2 with two functional groups showed much lower PCE (0.04%) mainly due to low solubility, as shown by microscopy studies below. We observed a huge shunt resistance and high serial resistance causing decrease of all electrical parameters. A third type of fullerene derivatives, [C60]P1 and [C70]P1, also revealed low solubility and provided very low PCEs, determined to be 0.018% and 0.007%, respectively, along with low *V_OC_*. Moreover, [C60]P1 showed s-shape IV characteristics with high serial resistance comparable with shunt resistance, which decreased the fill factor. In the case of [C70]P1 cells, huge shunt resistance was observed accompanied by a high serial resistance, which suggested low electric conductivity of this material. A similar adverse effect of the pyrene substituent on fulleroid solubility and, hence, on photovoltaic performances of inverted planar perovskite solar cells has recently been observed [37].

#### 2.2.2. Optical Absorption Spectroscopy

**Figure 6:**
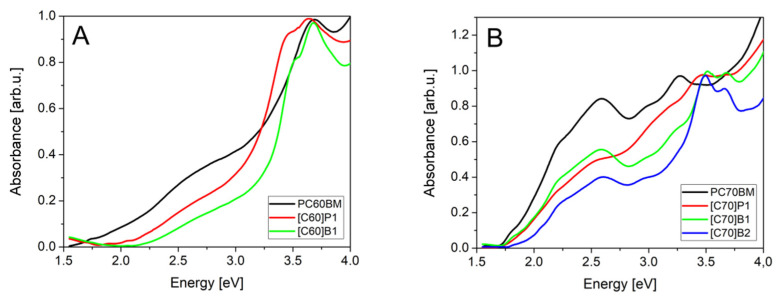
Absorption spectra of investigated fullerene derivatives: (**A**) C_60_, (**B**) C_70_.

In the measured spectral range normalized absorbance spectra of pure C_60_ fullerene derivatives (Figure 6A) showed that, for commercial material PC60BM, absorption started to grow at 1.6 eV and reached maximum at 3.6 eV. For [C60]P1 fullerene derivatives, we observed absorption threshold at higher energy, 1.8 eV, the main maximum peak was red shifted about 100 meV, and a new absorbance peak appeared at 3.3 eV. Additionally, for [C60]B1 a new absorption peak was observed, but at higher energy, 3.4 eV. The main absorption peak was in this case at the same energy as for PC60BM, but it was much narrower. The [C60]B1 absorption threshold was observed at energy of about 2.0 eV, higher than for other compounds.

Absorption spectra of C_70_ fullerene derivatives in Figure 6B showed that the standard material PC70BM had threshold at 1.7 eV and reached first maximum at 2.6 eV. These two features remained the same for all C_70_ fullerene derivatives and differences were visible only for the strongest second absorbance peak in the energy range 3.2–3.8 eV. The PC70BM had second absorption peak at 3.25 eV. The absorption threshold at about 1.7–1.8 eV is due to the transitions from the ground singlet S_0_ state to the S_1_-derived states [38]. They do not form peaks in the spectra due to very weak oscillator strength. The peak at 2.6 eV in C_70_ spectra is related to triplet transition S_0_ → T_1_, allowed by lower symmetry of the C_70_ molecule.

The peaks observed in 3.3–3.7 eV range are characteristic for different derivatives, so we expect that they are related to the pyrene substituents. However, it can be noticed that the peaks are single in the case of C_60_ derivatives and double in the case of C_70_ derivatives. Therefore, these are most probably transitions from states localized at the fullerene core, to states localized on the pyrene substituents.

For [C70]P1, all absorption peaks were much wider than for the rest of derivatives. The [C70]B1 and [C70]B2 had much narrower peaks and their spectra were similar to each other, probably due to the contents of the same functional group. They differed only in oscillator strength of the third absorption peaks.

We expect that the small differences between absorption of standard PCBM (both C_60_ and C_70_ derivatives) and new fullerene derivatives did not significantly change absorption of solar radiation. From the spectra, we conclude that the fullerenes [C60]P1 and [C60]B1 had higher energy gap so they absorb less light in 1.5–2.0 eV solar spectral range, while absorb more light in in the 3.0–3.5 eV range due to the additional absorption peak. The [C70]P1, [C70]B1 and [C70]B2 fullerenes had the second absorption peak shifted to higher energy where solar intensity is unfortunately lower.

**Figure 7:**
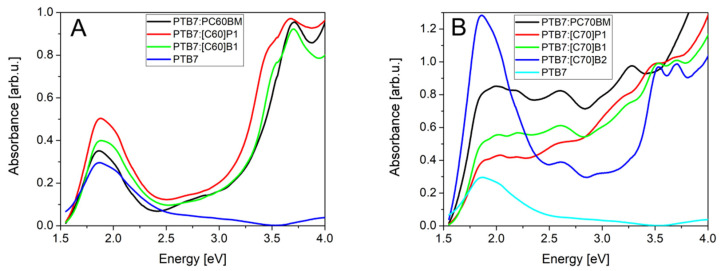
Absorption spectra of PTB7: fullerene derivatives: (**A**) C_60_, (**B**) C_70_.

Figure 7 presents the absorption spectra of PTB7: fullerene mixtures identical as in active layers of solar cells. Donor:acceptor ratio in the case of C_60_ derivatives was constant. The PTB7 donor material absorbs light mainly in 1.5 eV–2.5 eV spectral range with strongest absorption peak at 1.9 eV. For the fullerene derivatives, one can observe the additional peaks in the 3.0 eV–4.0 eV range, the same as for pure fullerene derivatives. Unfortunately, solar radiation in this range is weak, so gain of electron generation is small. Spectra of the PTB7 mixtures with [C70]P1, [C70]B1 and [C70]B2 showed second absorbance peak of fullerene in high energy range (about 3.5 eV). Donor:acceptor ratio in the case of C_70_ fullerene derivatives was probably different than designed due to low solubility (especially for [C70]B2) and high CS_2_ solvent evaporation rate. We could, therefore, expect that PTB7:[C70]P1 and PTB7:[C70]B1 had higher concentration of fullerene in active layer than PTB7:[C70]B2.

#### 2.2.3. Photoluminescence Spectroscopy

**Figure 8:**
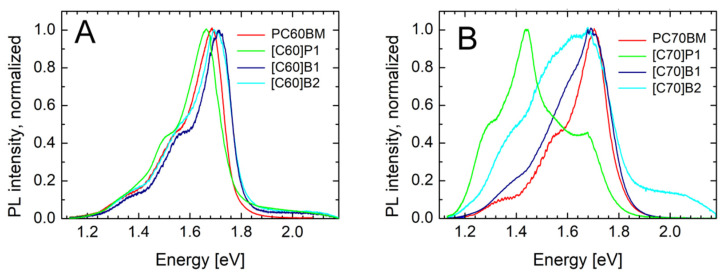
Luminescence spectra of the fullerene derivatives: (**A**) C_60_, (**B**) C_70_.

As can be observed in Figure 8A, PL spectra of the C_60_ derivatives contained mainly one peak at about 1.7 eV (730 nm). In literature, a similar peak has been related to singlet S1 → S0 transition in fullerene C_60_ [39]. The peaks of lower energies connected to vibronic transitions were at about 1.55 eV and 1.4 eV. The maxima of the S1 → S0 transition were at energies *hν* = 1.69 eV, 1.65 eV, 1.71 eV and 1.71 eV, for PC60BM, [C60]P1, [C60]B1, and [C60]B2, respectively.

The C_70_ derivatives PL spectra also had the peaks at about 1.7 eV. The maxima of the S1 → S0 transition were at energies *hν* = 1.70 eV, 1.69 eV, and 1.69 eV, for PC70BM, [C70]B1, and [C70]B2, respectively. However, there were also peaks at lower energies, in 1.4–1.5 eV (800–900 nm) range. They could be ascribed to triplet transition T1 → S0, allowed due to lower symmetry of the C_70_ molecule, or due to states localized at pyrene substituent. In the case of [C70]P1, this peak dominated the whole PL spectrum. Finally, it should be stated that the emission energies measured for the tested pure fullerene derivatives C_60_ and C_70_ are in good agreement with the results of the CV measurements. As shown in Figure 3, the values and shapes of both curves are similar.

The PTB7 luminescence forms of broad band in the range of 750 nm to 900 nm. In the case of the PTB7 mixtures with P1 (both C_60_ and C_70_ derivatives), the PTB7 luminescence was clearly visible. However, in the PTB7 mixtures with PCBM and B1 (both C_60_ and C_70_ derivatives), the PTB7 luminescence was completely dumped and also emission from these fullerene derivatives was much weaker (see Supplementary Materials, Figure S4). This was most probably due to fast and efficient separation of photo-excited carriers in the case of PTB7 mixtures with PC60BM, PC70BM and B1 fullerene derivatives. Obviously, this suggested also that the carrier separation was less efficient in the case of PTB7:P1 (both C_60_ and C_70_ derivatives). An additional support for this belief was the weak operation of solar cells based on these derivatives, as discussed above.

#### 2.2.4. Photocurrent Spectroscopy

Solar cells made with the use of the investigated fullerene derivatives as acceptor material mixed with PTB7 acting as donor material were measured by photocurrent spectroscopy. The obtained spectra of short circuit photocurrent, I_sc_(h*ν*) (measured in electrons/s), were divided by excitation intensity, Φ(h*ν*) (measured in photons/s), so the quantum external efficiency (EQE) spectra were obtained.

The average values of EQE followed the trend observed for power conversion efficiency (see Table 2): the highest values were observed for the cells with both PC60BM and PC70BM derivatives, B1 and P1 produced lower efficiencies, and B2 had very low output, all for both C_60_ and C_70_ derivatives. The average values of EQE for the cells containing C_60_ and C_70_ fullerene derivatives were similar but the shapes of the spectra were different. The values of the intensive part of the EQE spectra were about 25%, 1%, and 10%, for PCBM, P1, and B1, all for both C_60_ and C_70_ derivatives, respectively.

**Figure 9:**
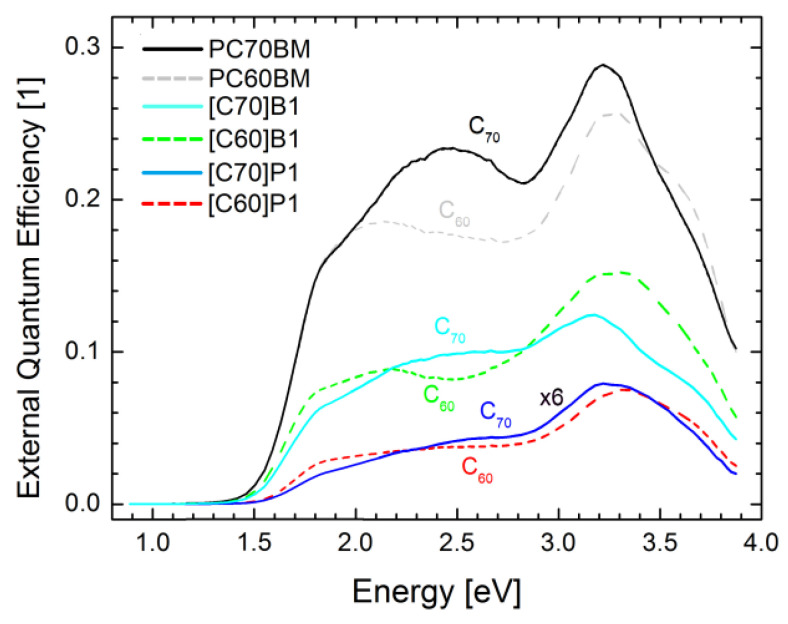
Comparison of EQE spectra of fullerene derivatives based on C_60_ and C_70_ fullerenes.

Such spectra contain usually the features from absorption spectra of both donor and acceptor materials [40]. The photocurrent spectra (see Figure 9) started from about 1.5 eV what is the absorption threshold of PTB7. At about 2.4 eV the PTB7 absorption decreases, and so EQE of the C_60_-containig cells decreased. However, in the case of the cells containing the C_70_ derivatives the EQE increased and the peaks about 2.6 eV were visible. Obviously these peaks were related to the characteristic fullerene C_70_ absorption peak at 2.6 eV observed in absorption (Figure 6B). This means that light absorbed in fullerenes also produces electron-hole pairs and the holes are transferred from fullerene to PTB7 generating photocurrent.

#### 2.2.5. Optical Microscopy

Since the solar cells are covered with transparent material, they can be easily examined by optical microscopy. The pictures below show the active areas of working solar cells.

**Figure 10:**
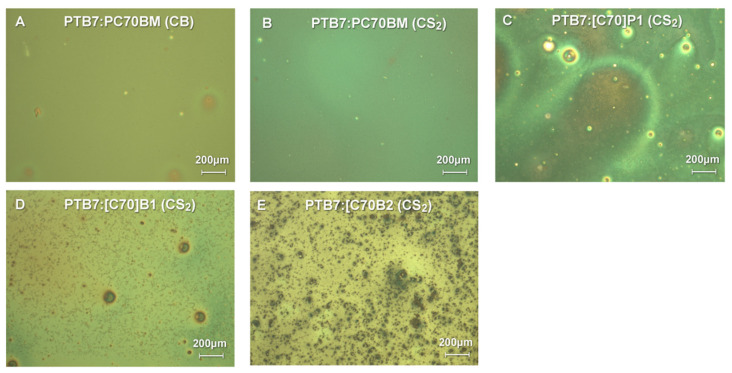
Optical microscopy images with differential interference contrast of solar cells prepared using PTB7 and synthesized C_60_/C_70_ pyrene derivatives and CS_2_ or CB as solvents.

Optical microscopy studies of PTB7:C70 active layers (Figure 10) indicated that the layers of the best quality layers were obtained for PTB7:PC70BM mixtures from both CB or CS_2_ solvents. Microscopic images showed homogenous layers with minor amounts of small solid inclusions. For the PTB7:[C70]B1 mixture, layers of a slightly lower quality were obtained, as wire-like inclusions and bigger solid material dots were observed on the surface produced on spincoated layers. The B1-derivatives (both C_60_ and C_70_ derivatives) were the best from the investigated here new fullerene derivatives and they worked relatively efficiently as photovoltaic active material in the solar cells. It was observed that PTB7:[C70]P1 and PTB7:[C70]B2 had the worst layer quality, with large inclusions of solid fullerene derivatives precipitated when applying the layer by spincoating. It coincided with their very low efficiency of the corresponding solar cells.

## 3. Materials and Methods

### 3.1. Reagents

Dichloromethane (DCM), toluene, n-hexane, ethyl malonyl chloride, malonyl chloride, 1-pyrenemethanol, 1-pyrenecarboxaldehyde, sarcosine, iodine, sodium and potassium were purchased from Sigma-Aldrich Poland (Poznań, Poland). 1,8-Diazabicycloundec-7-ene (DBU), and silica gel 70–230 mesh were obtained from Alfa Aesar (Ward Hill, MA, USA). C_60_ and C_70_ fullerenes were purchased from Nano-C (Westwood, MA, USA). Triethylamine, n-pentane and ethyl acetate were purchased from POCh (Gliwice, Poland). Toluene was dried over sodium and benzophenone. Other solvents were ACS grade and were used as received. PTB7, PC60BM, PC70BM, PEDOT:PSS (in AL 4083 water solution) and ITO 8 pixel glass substrates were purchased from Ossila (Sheffield, UK).

### 3.2. Synthesis of Malonate Esters

Pyrene malonic acid esters: were synthesized according to the method reported by de la Torre et al. [41]. Their spectral and chemical characterization can be found in Supplementary Materials (Figures S5–S12).

### 3.3. Synthesis of Methanofullerenes

Desired C_60_/C_70_ fullerene malonates were synthesized according to the modified Bingel method [17,42]. To a solution of corresponding fullerene (0.2 mmol) in anhydrous toluene (115 mL), malonate derivative (0.1 mmol), iodine (25 mg) dissolved in toluene (10 mL) and DBU (31 µL, 0.2 mmol) diluted with 5 mL of toluene were added. Resulting mixture was stirred overnight at room temperature under argon atmosphere. After concentration under reduced pressure, the obtained mixture was chromatographed (silica gel 70–230 mesh, toluene/n-hexane 2:1) to yield fullerene pyrene malonates as brown or blackish powders. Detailed analysis of synthesized fullerene derivatives can be found in Supplementary Materials (Figures S13–S26).

### 3.4. Synthesis of Fulleropyrrolidines

Pyrene substituted fulleropyrrolidines were obtained using the modified procedure reported by Prato et al. [16]. To a well-stirred solution of corresponding fullerene (0.2 mmol) in freshly distilled toluene (130 mL), corresponding aldehyde (0.1 mmol) and sarcosine (89 mg, 1 mmol) were added. Resulting mixture was refluxed under argon atmosphere for 8 h. The obtained reaction mixture was then allowed to cool down to room temperature, and filtered to remove insoluble residue. Then, it was concentrated under reduced pressure and chromatographed on silica gel using mixture of *n*-hexane and toluene (1:2 for C_60_ derivatives and 1:4 for functionalized C_70_ fullerene). Fractions containing the desired products were concentrated under vacuum, precipitated with n-pentane and dried in vacuum to give pure fulleropyrrolidines dark brown/blackish powders. In case of C_70_ fulleropyrrolidine the resulting regioisomers were not separated. Detailed characterization of the synthesized fulleropyrrolidines can be found in Supplementary Materials (Figures S27–S32).

### 3.5. Methods

Theoretical calculations were performed using the Gaussian 16 software package [43]. The density functional theory (DFT) method with the B3LYP hybrid functional [44,45,46] and the 6-31G(d) basis set was used for the geometry optimization of the investigated fullerene derivatives. The energy gaps between the lowest unoccupied molecular orbital (LUMO) energy and the highest occupied molecular orbital (HOMO) energy were calculated on fully optimized structures at the DFT/B3LYP/6-31G(d) level by employing both the DFT/B3LYP 6-31G(d) and DFT/PBE/6-311G(d,p) approach.

ESI-MS spectra were recorded on a Micromass LCT ESI-TOF mass spectrometer equipped with an orthogonal electrospray ionization source (Wilmslow, UK). ^1^H- and ^13^C-NMR spectra were acquired on Varian Unity Plus 500 MHz spectrometer using CDCl_3_ as a solvent (Palo Alto, CA, USA). The infrared measurements were performed using Shimadzu FTIR-8400S (Kioto, Japan). UV-Vis spectra were collected in quartz glass cuvette using Varian Carry 50 UV-Vis spectrophotometer and Carry 5000 UV-Vis-NIR spectrophotometer in the spectral range from 200 to 800 nm (Palo Alto, CA, USA).

Optical microscopy images of solar cell active layer between ITO and Al electrodes were obtained by Nikon ECLIPSE ME600 microscope with differential interference (Nomarski) contrast and DeltaPix digital camera (Tokio, Japan). Objective with x5 magnification was used.

Cyclic voltammetry (CV) measurements were carried out in a conventional three-electrode cell, with a Pt disc of the surface area of 0.03 cm^2^ as the working electrode, a Pt wire as the counter electrode and a double junction Ag/Ag^+^ (0.1 M in AN, with a salt bridge filled with 0.1 M TBAPF_6_ solution in DCM) as the reference electrode. Since stability of the potential of this electrode is sometimes not good enough, the ferrocene/ferrocenium (Fc/Fc^+^) couple was added to the solutions after each measurement. Thus, the potential in the plots was quoted to the formal potential of Fc/Fc^+^ redox couple, which was of about 0.117 V vs. Ag/Ag^+^ electrode and 0.464 V vs. standard hydrogen electrode (SHE).

The cyclic voltammograms were performed in DCM solutions containing fullerene derivatives and 0.1 M TBAPF_6_ supporting electrolyte. All solutions before experiments were deoxygenated by bubbling with a stream of Ar for 10 min. The Pt electrode before each use was polished with a polycrystalline diamond suspension (3 μm) (MetaDiTM Supreme, Buehler) on a polishing cloth. 

The photoluminescence (PL) measurements were performed using frequency-tripled output pulses (130 fs, wavelength 300 nm) from a mode-locked Ti:sapphire laser. The spectral distribution of the PL was analyzed by an ACTON320 spectrometer (1 meV resolution). For the photoluminescence (PL) spectroscopy, the samples were prepared by casting of the solutions on a copper plate and drying at room temperature. Pure fullerene derivatives and their mixtures with PTB7 were measured.

Photocurrent spectroscopy and external quantum efficiency measurements were performed in the spectral range of 320 to 1400 nm (0.8 to 3.9 eV), using tungsten lamp with a monochromator. The current was measured by Keithley picoammeter and light intensity spectra were obtained with use of PM320 power-meter.

Current-voltage characteristics of the solar cells were measured by KEITHLEY 2450 Source Meter with Kickstart PC software (v2.4.0, Tektronix: Beaveton, OR, USA, 2020), and using Ossila 8-pixel test board. Newport VeraSol-2 LED Class AAA Solar Simulator of 1000 W/m^2^ power output and AM1.5G spectrum was applied as a source of illumination. Average PCE was calculated for 8 pixels of each cell (Irvine, CA 92606, USA).

### 3.6. Solar Cells Preparation

**Figure 11:**
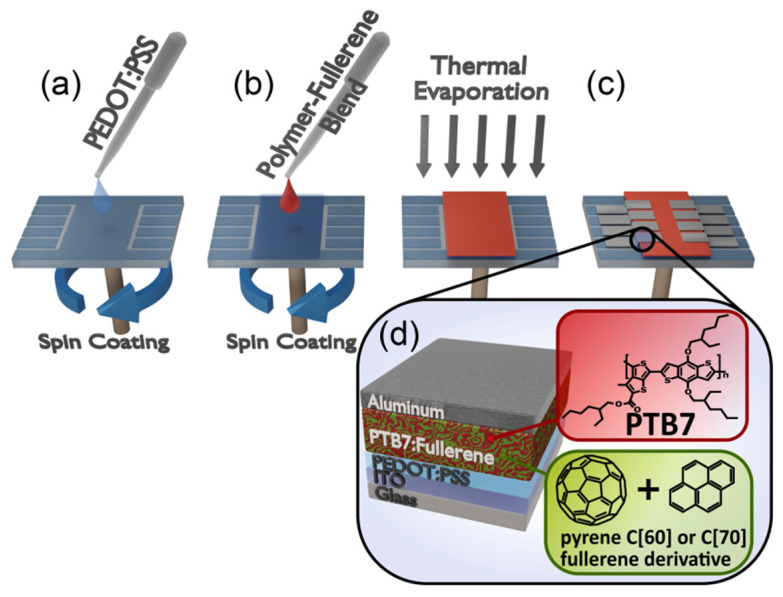
Solar cells preparation scheme (**a**) PEDOT:PSS solution is dynamically spin-coated onto ITO substrate to form an uniform coating layer, (**b**) Active layer solution is likewise spin-coated, (**c**) aluminum cathode is sputtered through mask to obtain 8 separated pixels, (**d**) final structure.

The investigated solar cells were based on conducting conjugated polymer PTB7 and fullerene derivatives (PC60BM, PC70BM, [C60]P1, [C70]P1, [C60]B1, [C70]B1, [C70]B2), forming bulk heterojunction (BHJ) for electron and hole generation and their separation (Figure 11). They were fabricated on the substrates covered with conductive and transparent indium-tin oxide (ITO). The substrates had 8 separated pixels with an area of 4 mm^2^ each. The efficient flow of holes and suppression of electron leakage to the ITO anode was secured by PEDOT:PSS electron blocking layer.

The reference PTB7:PC60BM and PTB7:PC70BM blends, both in 2:3 weight ratio, were dissolved in chlorobenzene (CB) or in carbon disulfide (CS_2_). Blends of PTB7 with the new fullerene derivatives were dissolved in CS_2_ due to their low solubility in chlorobenzene. Carbon disulfide has much higher evaporation rate at room temperature compared to chlorobenzene, so the control of the layer thickness during spincoating was difficult. Instead, we controlled the layer thickness by changing the total concentration of solutions. The ideal total concentration of PTB7:fullerene solution in CS_2_ turned out to be 12 mg/mL, while the total concentration of reference in CB was 25 mg/mL. The PTB7:fullerene derivative weight ratio of was 2:3 for all samples. Solutions in carbon disulfide were prepared on air and stirred for 1 h at room temperature before deposition. The reference solutions were prepared in argon glove box and stirred for 24 h at 50 °C. Before deposition, the solutions were cooled down and filtered through 0.45 μm PTFE syringe filter.

The glass substrates cover with ITO were cleaned in Ossila UV Ozone Cleaner (Sheffield, UK) for 5 min and then PEDOT:PSS layer was deposited by spincoating in air (5000 rpm, 60 s) and annealed for 15 min at 150 °C. Then active layer was spincoated on top of the PEDOT:PSS layer. The reference PTB7:PCBM solutions in chlorobenzene were spincoated (900 rpm, 60 s) in a glovebox. The PTB7:new-fullerene derivatives solutions in carbon disulfide were spincoated (2000 rpm, 60 s) in air. For both types of active layer solutions, the deposited active layers were dried in vacuum (1 mbar) for 15 min to remove residual solvent. Finally, the Al cathode was thermally evaporated on the structure.

Samples of organic layers for absorption measurements were spincoated on pure glass substrates. Solutions of PTB7:fullerene derivative active layers were prepared and spincoated using the same procedure as for the active layers of solar cells. In the case of pure materials, solutions of PTB7, PC60BM, PC70BM in chlorobenzene and the solutions of [C60]P1, [C70]P1, [C60]B1, [C70]B1, [C70]B2 in carbon disulfide were prepared with concentration of 15 mg/mL.

## 4. Conclusions

A series of C_60_ and C_70_ pyrene derivatives of fullerene was synthesized and subjected to thorough theoretical studies using DFT to determine their energy levels in the HOMO and LUMO orbitals. The theoretical results were compared with the experimental data obtained with the cyclic voltammetry. It turned out that the DFT/B3LYP/6-31G (d) approach reflects the experimental HOMO orbital better than the DFT/PBE/6-311G (d, p) method, while the latter method very accurately reflects the energies of the LUMO orbital. For the synthesized pyrene derivatives of fullerene C_60_ and C_70_, the model LUMO orbitals are always found on the fullerene ball while orbital HOMO are only located on pyrene moieties. The PC60BM and PC70BM tested for comparison are characterized by the fact that both HOMO and LUMO orbitals are located on the fullerene sphere.

Both the experimental results of the CV and theoretical estimates obtained by means of quantum-mechanical calculations indicate that for the tested mono- and di-pyrene derivatives, the type of the link to the cage and the number of pyrene residues forming a substituent on C_60_ and C_70_ fullerenes do not significantly affect the energy level of the HOMO and LUMO orbitals. A slightly greater range of energy changes is observed in the HOMO orbital. Thus, it seems that the differentiated efficiency of photovoltaic cells composed of their share must depend on the factors other than the electronic properties. Chlorobenzene, a commonly used solvent for the production of photosensitive layers for photovoltaic materials, could not be used to produce the solar cells based on them due to the negligible solubility of the tested fullerene materials. It was replaced with carbon disulfide, which improved the situation, but due to low enthalpy of vaporization, CS_2_ made it difficult to produce homogeneous photosensitive layers. We observed slight differences between the absorption of PCBM reference fullerene derivatives (both C_60_ and C_70_ derivatives) and new fullerene derivatives, which suggests that absorption is not a decisive factor in the efficiency of solar radiation absorption and, therefore, the reliability of the cells made of them.

The photoluminescence spectroscopy seems to explain why the derivatives obtained in the Prato P1 reaction are generally less effective than the B1 derivatives in effectively constructed photochemical devices. This is most probably due to slow and less efficient separation of photo-excited carriers in P1 derivatives. However, data from photocurrent spectroscopy measurements show that despite the low efficiency of the designed solar cells, the synthesized fullerene derivatives absorb light producing electron-hole pairs, and then holes are transferred from the fullerene to PTB7 generating photocurrent.

It seems, however, that the most important condition which determines the efficiency of the tested photovoltaic devices is the homogeneity of the photoactive layer composed of PTB7 and a fullerene derivative. The analysis of the images obtained by optical microscopy clearly shows that the less solid inclusions and the more homogeneous the layer is, the higher the power conversion efficiency is achieved.

## Figures and Tables

**Table 1:** HOMO and LUMO levels and LUMO-HOMO energy gaps (Eg) determined from the CV measurements.

Fullerene Derivative	LUMO (eV)	HOMO (eV)	Eg (eV)
[C60]P1	−3.88	−5.36	1.48
[C60]B1	−3.89	−5.56	1.67
[C60]B2	−3.95	−5.67	1.72
[C70]P1	−3.85	−5.49	1.64
[C70]B1	−3.83	−5.58	1.75
[C70]B2	−3.86	−5.57	1.71

**Table 2:** Electrical parameters of solar cells constructed using investigated C_60_ and C_70_ fullerene derivatives. Voc-open-circuit volage, Jsc-short circuit current density, FF-fill factor, Rsh and RS-shunt and serial resistivities, max PCE and avg PCE-maximal and averaged (over 8 pixels) PCE.

Sample	*V_OC_* (V)	J_SC_ (mA/cm^2^)	FF (%)	Rs (Ω·cm^2^)	Rsh (Ω·cm^2^)	Max PCE (%)	Av. PCE (%)
PTB7:PC_60_BM (CB)	0.696	9.28	55.6	5.24	581	3.59	3.31
PTB7:PC_60_BM (CS_2_)	0.725	5.24	60.4	4.37	681	2.30	2.04
PTB7:[C60]P1 (CS_2_)	0.306	0.29	20.9	610	785	0.018	0.011
PTB7:[C60]B1 (CS_2_)	0.697	2.62	41.0	32.7	485	0.75	0.45
PTB7:PC_70_BM (CB)	0.711	11.5	56.4	5.45	501	4.59	3.95
PTB7:PC_70_BM (CS_2_)	0.733	5.91	57.5	4.38	468	2.49	2.28
PTB7:[C70]P1 (CS_2_)	0.225	0.09	33.5	23.3	3220	0.007	0.004
PTB7:[C70]B1 (CS_2_)	0.713	2.49	50.7	26.9	724	0.90	0.83
PTB7:[C70]B2 (CS_2_)	0.433	0.34	28.6	98.2	1370	0.043	0.037

## Data Availability

All data is contained within the article or Supplementary Materials.

## Supplementary Materials

The following are available online. Figures S1 and S2: Cyclic voltammograms of [C60]B1, [C70]B1, [C60]P1 and [C70]P1, Figure S3: Optimized structures of the four isomers of [C70]P1, Figure S4: PL spectra of PTB7 and the blends containing PTB7 and different fullerene derivatives, Figures S5–S32: ESI-MS, ^1^H-NMR, ^13^C-NMR and FT-IR spectra of synthesized compounds. Table S1: Optimized Geometries at DFT B3LYP/6-31g(d) level.

## Sample Availability

Samples of the compounds are not available from the authors.

## References

[1] Li C.-Z., Yip H.-L., Jen A.K.-Y. (2012). Functional fullerenes for organic photovoltaics. J. Mater. Chem..

[2] Ganesamoorthy R., Govindasamy S., Sakthivel P. (2017). Review: Fullerene based acceptors for efficient bulk heterojunction organic solar cell applications. Sol. Energy Mater. Sol. Cells.

[3] Kim H.U., Kim J.-H., Kang H., Grimsdale A.C., Kim B.J., Yoon S.C., Hwang D.-H. (2014). Naphthalene-, anthracene-, and pyrene-substituted fullerene derivatives as electron acceptors in polymer-based solar cells. ACS Appl. Mater. Interfaces.

[4] Cominetti A., Pellegrino A., Longo L., Po R., Tacca A., Carbonera C., Salvalaggio M., Baldrighib M., Meille S.V. (2015). Polymer solar cells based on poly(3-hexylthiophene) and fullerene:pyrene acceptor systems. Mater. Chem. Phys..

[5] Gareis T., Köthe O., Daub J. (1998). Chromophore-appended C_60_ and C_70_ fullerenes: Anthracene and pyrene derivatives—Syntheses, cyclic voltammetry, and spectra. Eur. J. Org. Chem..

[6] Dallas P., Rogers G., Reid B., Taylor R.A., Shinohara H., Briggs G.A.D., Porfyrakis K. (2016). Charge separated states and singlet oxygen generation of mono and bis adducts of C_60_ and C_70_. Chem. Phys..

[7] Zaragoza-Galán G., Ortíz-Palacios J., Valderrama B.X., Camacho-Dávila A.A., Chávez-Flores D., Ramos-Sánchez V.H., Rivera E. (2014). Pyrene-fullerene C_60_ dyads as light-harvesting antennas. Molecules.

[8] Kathiravan A., Panneerselvam M., Sundaravel K., Pavithra N., Srinivasan V., Anandand S., Jaccob M. (2016). Unravelling the effect of anchoring groups on the ground and excited state properties of pyrene using computational and spectroscopic methods. Phys. Chem. Chem. Phys..

[9] Tang C., Liu F., Xia Y.-J., Lin J., Xie L.-H., Zhong G.-Y., Fan Q.-L., Huang W. (2006). Fluorene-substituted pyrenes—Novel pyrene derivatives as emitters in non-doped blue OLEDs. Org. Electron..

[10] Jeong S.-H., Lee K.H., Lee J.Y. (2019). Dual role of a pyrene derivative as a hole transport material and an emitter in blue fluorescent organic light-emitting diodes. Dye. Pigment..

[11] Xu J.-Q., Liu W., Liu S.-Y., Ling J., Mai J., Lu X., Li C.-Z., Jen A.K.-Y., Chen H. (2017). A-D-A small molecule donors based on pyrene and diketopyrrolopyrrole for organic solar cells. Sci. China Chem..

[12] Carati C., Gasparini N., Righi S., Tinti F., Fattori V., Savoini A., Cominetti A., Po R., Bonoldi L., Camaioni N. (2016). Pyrene−fullerene interaction and its effect on the behavior of photovoltaic blends. J. Phys. Chem. C.

[13] Alrawashdeh A.I., Zhao Y., Lagowski J.B. (2019). Conformational analysis of the supramolecular complexation of diaryl-substituted tetrathiafulvalene vinylogues with fullerenes. ACS Omega.

[14] Lin H., Du X., Li L., Zheng C., Tao S. (2018). Pyrene-imidazole based aggregation modifier leads to enhancement in efficiency and environmental stability for ternary organic solar cells. Front. Chem..

[15] Zhang J., Li C.-Z., Williams S.T., Liu S., Zhao T., Jen A.K.-Y. (2015). Crystalline co-assemblies of functional fullerenes in methanol with enhanced charge transport. J. Am. Chem. Soc..

[16] Prato M., Maggini M. (1998). Fulleropyrrolidines:  A family of full-fledged fullerene derivatives. Acc. Chem. Res..

[17] Bingel C. (1993). Cyclopropanierung von Fullerenen. Chem. Ber..

[18] Beu T.A., Onoe J., Hida A. (2005). First-principles calculations of the electronic structure of one-dimensional C_60_ polymers. Phys. Rev..

[19] Benallal R., Boughrraf H., Sahdane T., Essassi E.M., Kabouchi B. (2017). Theoretical and experimental investigations of structural and electronic properties of 1-Benzyl-3-methyl-quinoxalin-2(1H)-one molecule. J. Mater. Environ. Sci..

[20] Reiss H., Heller A. (1985). The absolute potential of the standard hydrogen electrode: A new estimate. J. Phys. Chem..

[21] Gaspar H., Figueira F., Strutyński K., Melle-Franco M., Ivanou D., Tomé J.P.C., Pereira C.M., Pereira L., Mendes A., Viana J.C. (2020). Thiophene- and carbazole-substituted n-methyl-fulleropyrrolidine acceptors in PffBT4T-2OD based solar cells. Materials.

[22] Zhang X., Li X.-D. (2014). Effect of the position of substitution on the electronic properties of nitrophenyl derivatives of fulleropyrrolidines: Fundamental understanding toward raising LUMO energy of fullerene electron-acceptor. Chin. Chem. Lett..

[23] Berger P.R., Kim M. (2018). Polymer solar cells: P3HT:PCBM and beyond. J. Renew. Sustain. Energy.

[24] He Z., Xiao B., Liu F., Wu H., Yang Y., Xiao S., Wang C., Russell T.P., Cao Y. (2015). Single-junction polymer solar cells with high efficiency and photovoltage. Nat. Photonics.

[25] Dang M.T., Hirsch L., Wantz G. (2011). P3HT:PCBM, best seller in polymer photovoltaic research. Adv. Mater..

[26] Kumar A., Baccoli R., Fais A., Cincott A., Pilia L., Gatto G. (2020). Substitution effects on the optoelectronic properties of coumarin derivatives. Appl. Sci..

[27] Peverati R., Truhlar D.G. (2014). Quest for a universal densityfunctional: The accuracy ofdensity functionals across abroad spectrum of databasesin chemistry and physics. Phil. Trans. R. Soc. A.

[28] Mohajeri A., Omidvara A. (2015). Fullerene-based materials for solar cell applications: Design of novel acceptors for efficient polymer solar cells—A DFT study. Phys. Chem. Chem. Phys..

[29] Zhou B., Hu Z., Jiang Y., He X., Sun Z., Sun H. (2018). Benchmark study of ionization potentials and electron affinities of armchair single-walled carbon nanotubes using density functional theory. J. Phys..

[30] Wang H., He Y., Li Y., Su H. (2012). Photophysical and electronic properties of five PCBM-like C 60 derivatives: Spectral and quantum chemical view. J. Phys. Chem. A.

[31] Li Z.-J., Yang W.-W., Gao X. (2011). A room-temperature fluorescence study of organofullerenes: Cis-1 bisadduct with unusual blue-shifted emissions. J. Phys. Chem. A.

[32] Bredas J.-L. (2017). Organic electronics: Does a plot of the HOMO–LUMO wave functions provide useful information?. Chem. Mater..

[33] Peverati R., Truhlar D.G. (2011). Communication: A global hybrid generalized gradient approximation to the exchange-correlation functional that satisfies the second-order density-gradient constraint and has broad applicability in chemistry. J. Chem. Phys..

[34] Rostami Z., Hosseinian A., Monfared A. (2018). DFT results against experimental data for electronic properties of C 60 and C 70 fullerene derivatives. J. Mol. Graph. Model..

[35] Cowan S.R., Roy A., Heeger A. (2010). Recombination in polymer-fullerene bulk heterojunction solar cells. J. Phys. Rev. B.

[36] Dennler G., Scharber M.C., Brabec C.J. (2009). Polymer-fullerene bulk-heterojunction solar cells. Adv. Mater..

[37] Castro E., Artigas A., Pla-Quintana A., Roglans A., Liu F., Perez F., Lledó A., Zhu X.-Y., Echegoyen L. (2019). Enhanced open-circuit voltage in perovskite solar cells with open-cage [60]fullerene derivatives as electron-transporting materials. Materials.

[38] Su C.-F., Wang S.-S., Tang S.-J., Wang J.-S., Chiu K.-C. (2010). Physica B: Condensed matter. Phys. B Condens. Matter.

[39] Sibley S.P., Argentine S.M., Francis A.H. (1992). A photoluminescence study of C_60_ and C_70_. Chem. Phys. Lett..

[40] Korona K.P., Korona T., Rutkowska-Zbik D., Grankowska S., Iwan A., Kamińska M. (2015). Polyazomethine as a component of solar cells-theoretical and optical study. J. Phys. Chem. Solids.

[41] De la Torre M.D.L., Tomé A.C., Silva A.M.S., Cavaleiro J.A.S. (2002). Synthesis of [60]fullerene–quercetin dyads. Tetrahedron Lett..

[42] Nierengarten J.-F., Gramlich V., Cardullo F., Diedrich F. (1996). Regio- and diastereoselective bisfunctionalization of C_60_ and enantioselective synthesis of a C_60_ derivative with a chiral addition pattern. Angew. Chem. Int. Ed..

[43] Frisch M.J., Trucks G.W., Schlegel H.B., Scuseria G.E., Robb M.A., Cheeseman J.R., Scalmani G., Barone V., Petersson G.A., Nakatsuji H. (2016). Gaussian 16.

[44] Kohn W., Sham L.J. (1965). Self-consistent equations including exchange and correlation effects. Phys. Rev..

[45] Becke A.D. (1988). Density-functional exchange-energy approximation with correct asymptotic behavior. Phys. Rev. A.

[46] Lee C., Yang W., Parr R.G. (1988). Development of the Colle-Salvetti correlation-energy formula into a functional of the electron density. Phys. Rev. B.

