# Supplementary material for: Mono- and Di-Pyrene [60]Fullerene and [70]Fullerene Derivatives as Potential Components for Photovoltaic Devices
Source: Molecules. 2021 Mar 12;26(6):1561. doi: 10.3390/molecules26061561 (PMC7998167; doi:10.3390/molecules26061561)
Supplement: Supplementary file 1 [file molecules-26-01561-s001.pdf]

Supplementary Materials

Mono- and di-pyrene [60]fullerene and [70]fullerene derivatives as potential components for photovoltaic devices

Piotr Piotrowski,[1] Wojciech Mech,[2] Kamila Zarębska,[1] Maciej Krajewski,[2] Krzysztof P. Korona,[2] Maria Kamińska,[2] Magdalena Skompska[1] and Andrzej Kaim*[1]

[1] Faculty of Chemistry, University of Warsaw, Pasteura 1, 02-093, Warsaw, Poland
[2] Faculty of Physics, University of Warsaw, Pasteura 5, 02-093 Warsaw, Poland
* Correspondence: akaim@chem.uw.edu.pl

Determination of HOMO and LUMO levels from CV measurements.

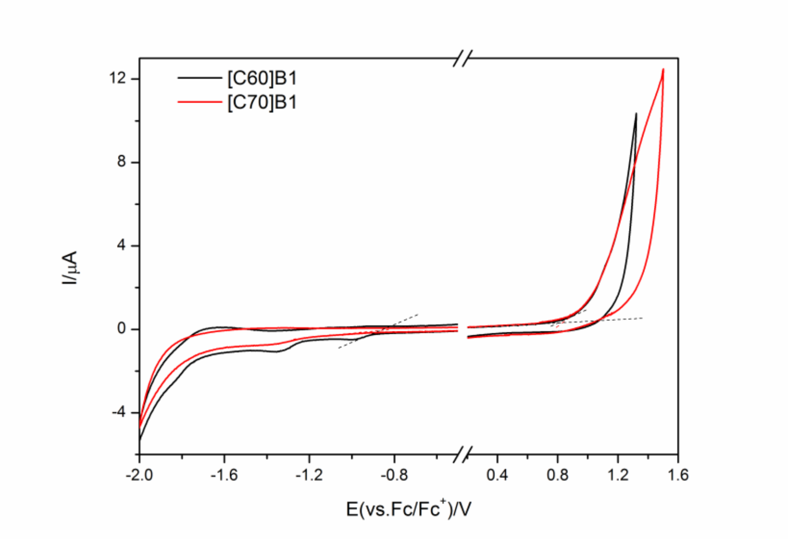

Figure S1. Cyclic voltammograms on Pt electrode in the solutions of [C60]B1 (1) and [C70]B1 (2) in CH_2Cl_2 containing 0.1 M $TBAPF_6$ supporting electrolyte.

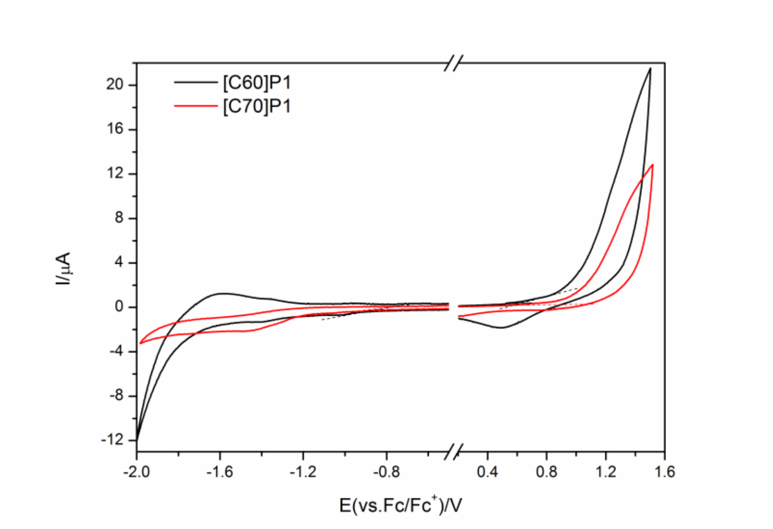

Figure S2. Cyclic voltammograms on Pt electrode in the solutions of [C60]P1 (1) and [C70]P1 (2) in CH_2Cl_2 containing 0.1 M $TBAPF_6$ supporting electrolyte.

Theoretical HOMO and LUMO frontier orbitals by PBE and BLYP functional calculations.

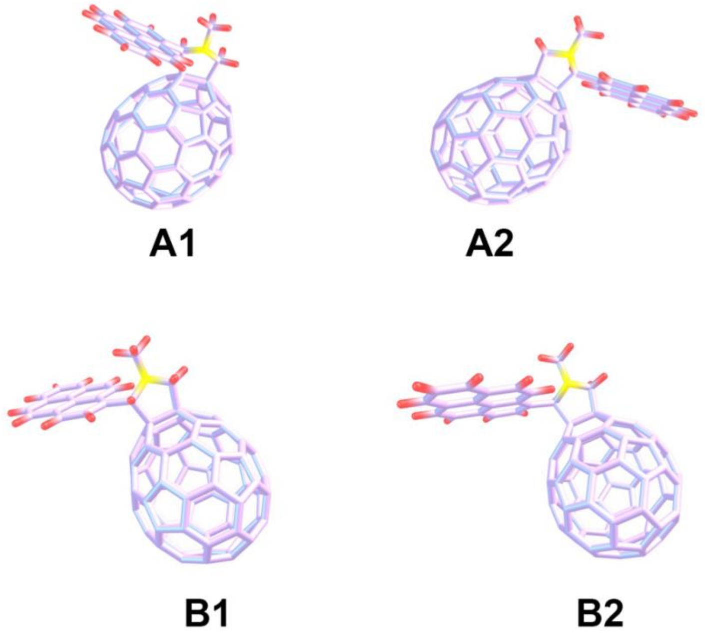

Figure S3. Optimized structures of the four isomers of **[C70]P1.**[1]

Photoluminescence spectroscopy

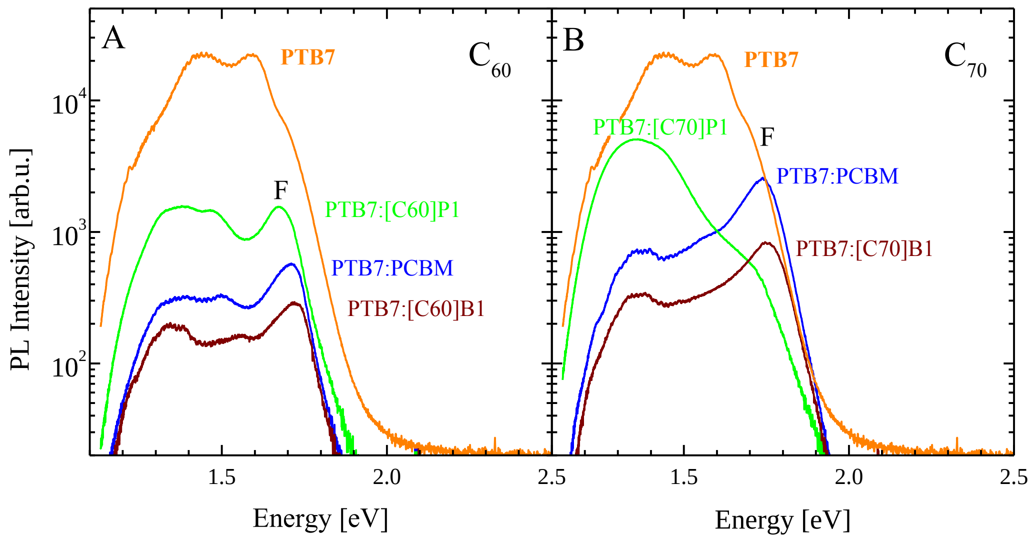

Figure S4. PL spectra of PTB7 and the blends containing PTB7 and different fullerene derivatives (cast from CS2 solution) A) C60 B) C70. Peak F is characteristic for fullerene. It can be observed that PTB7 luminescence is strongly dumped (even 100 times). However, dumping depends on derivative. It is strongest in the case of B1 and weakest in the case of P1.

[1]H. Gaspar, F. Figueira, K. Strutyński, M. Melle-Franco, D. Ivanou, J.P.C. Tomé, C.M. Pereira, L. Pereira, A. Mendes, J.C. Viana and G. Bernardo, *Materials*, 2020, **13**, 1267.

Synthesis of malonate esters

Ethyl (pyren-1-ylmethyl) malonate (MP):

Yield: 88%; mp = 62.9°C; ESI-MS: 368.9 $[M+Na]^+$; FT-IR (KBr) V_{max} (cm^{-1}) 3041.42, 2990.70, 2907.53, 1738.37, 1720.41, 1472.52, 1412.87, 1374.82, 1364.44, 1334.95, 1289.62, 1186.97, 1161.42, 1154.95, 1113.77, 1033.49, 977.17, 844.18, 839.89, 755.14, 722.96, 709.70, 620.32; ^1H NMR (500 MHz, $CDCl_3$) δ 8.29-8.00 (m, 9H), 5.91 (s, 2H), 4.14 (q, *J*=7.1Hz, 2H), 3.44 (s, 2H), 1.16 (t, *J*=7.1Hz, 3H)ppm; ^{13}C NMR (125MHz, $CDCl_3$) δ 166.62, 166.41, 131.90, 131.17, 130.65, 129.59, 128.29, 128.09, 127.94, 127.88, 127.32, 126.11, 125.58, 125.51, 124.85, 124.59, 122.81, 65.75, 61.58, 41.67, 13.92ppm;

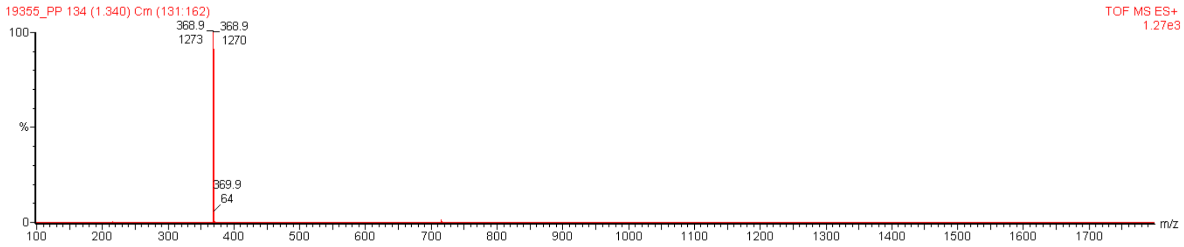

Figure S5. ESI-MS spectrum of ethyl (pyren-1-ylmethyl) malonate (MP).

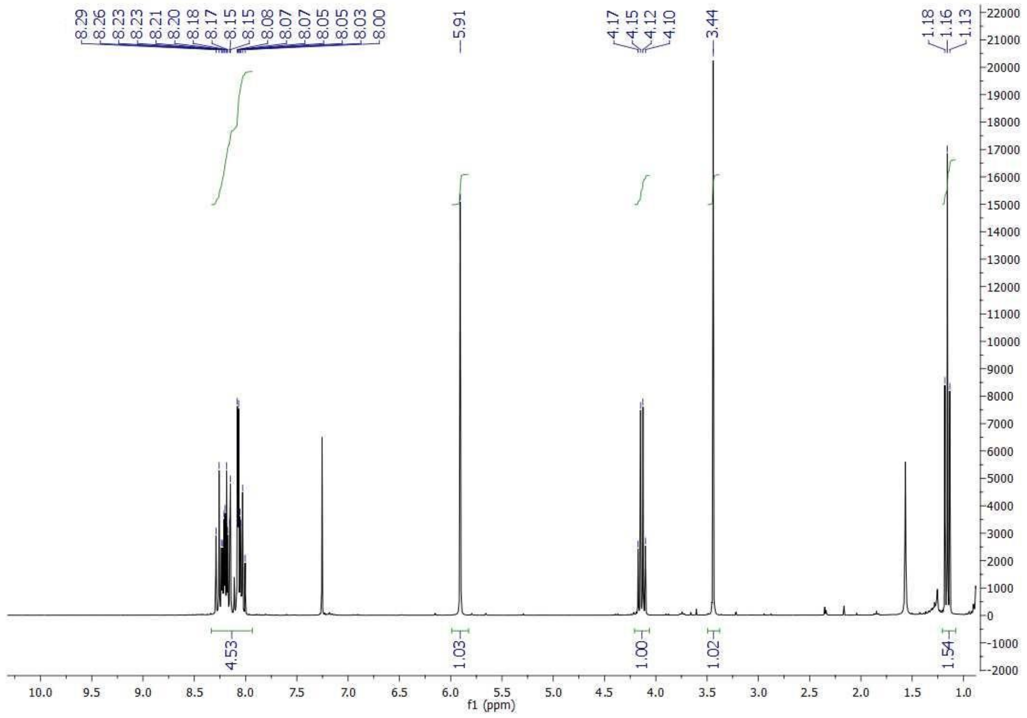

Figure S6. ^1H NMR spectrum of ethyl (pyren-1-ylmethyl) malonate (MP).

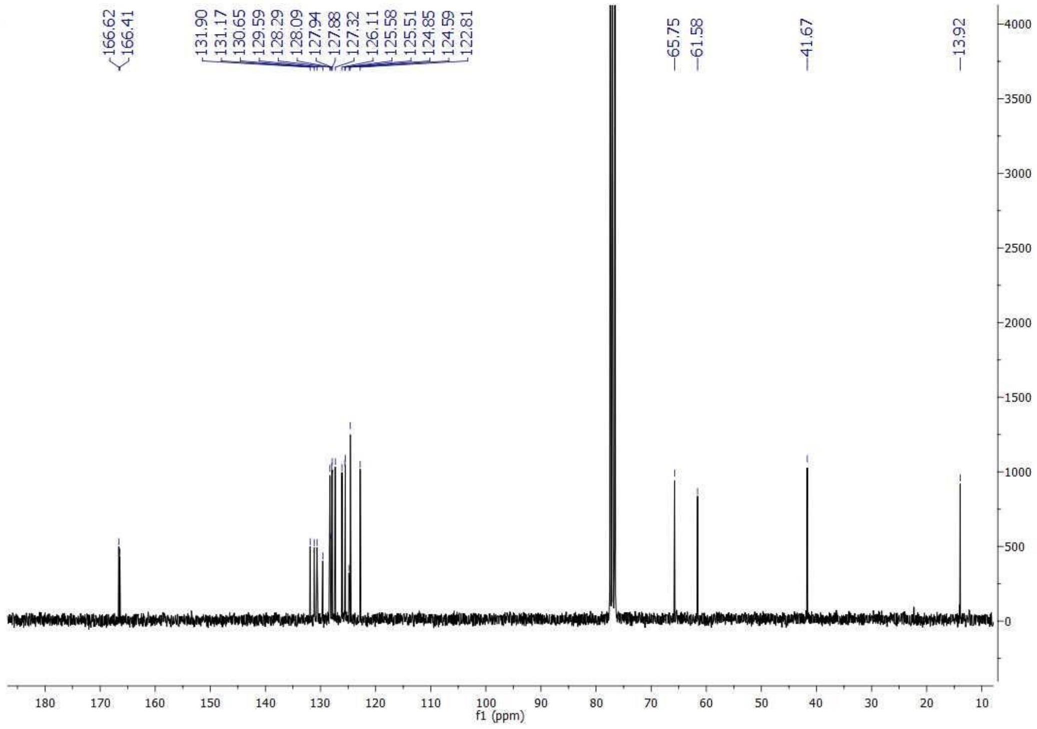

Figure S7. ^{13}C NMR spectrum of ethyl (pyren-1-ylmethyl) malonate (MP).

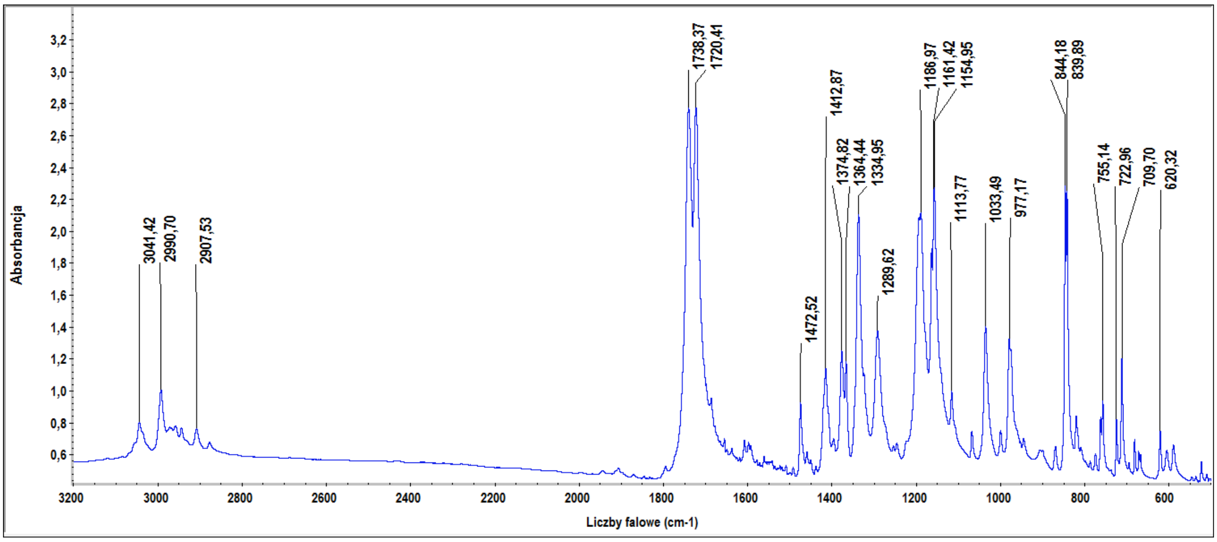

Figure S8. FT-IR spectrum of ethyl (pyren-1-ylmethyl) malonate (MP).

Bis(pyren-1-ylmethyl) malonate (BP):

Yield: 78%; mp = 166.6°C; ESI-MS: 555.0 $[M+Na]^+$; FT-IR (KBr) V_{max} (cm^{-1}) 3040.10, 2961.90, 2921.69, 1733.44, 1720.78, 1458.29, 1418.07, 1382.55, 1335.20, 1321.70, 1220.93, 1190.47, 1161.83, 1065.07, 984.26, 970.86, 931.02, 879.63, 848.14, 840.09, 821.55, 751.29, 715.44, 705.37, 680.79, 620.97; ^1H NMR (500 MHz, $CDCl_3$) δ 8.16-7.87 (m, 9H), 5.82 (s, 2H), 3.54 (s, 1H) ppm; ^{13}C NMR (125MHz, $CDCl_3$) δ 166.41, 131.77, 131.11, 130.57, 129.39, 128.23, 127.87, 127.58, 127.28, 126.03, 125.51, 125.45, 124.50, 122.62, 65.80, 41.82ppm;

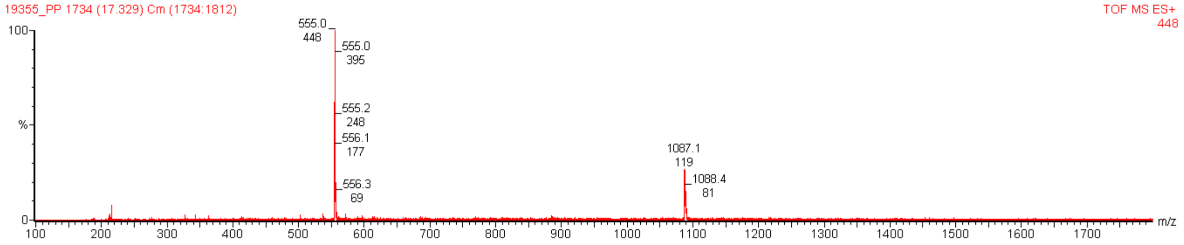

Figure S9. ESI-MS spectrum of bis(pyren-1-ylmethyl) malonate (BP).

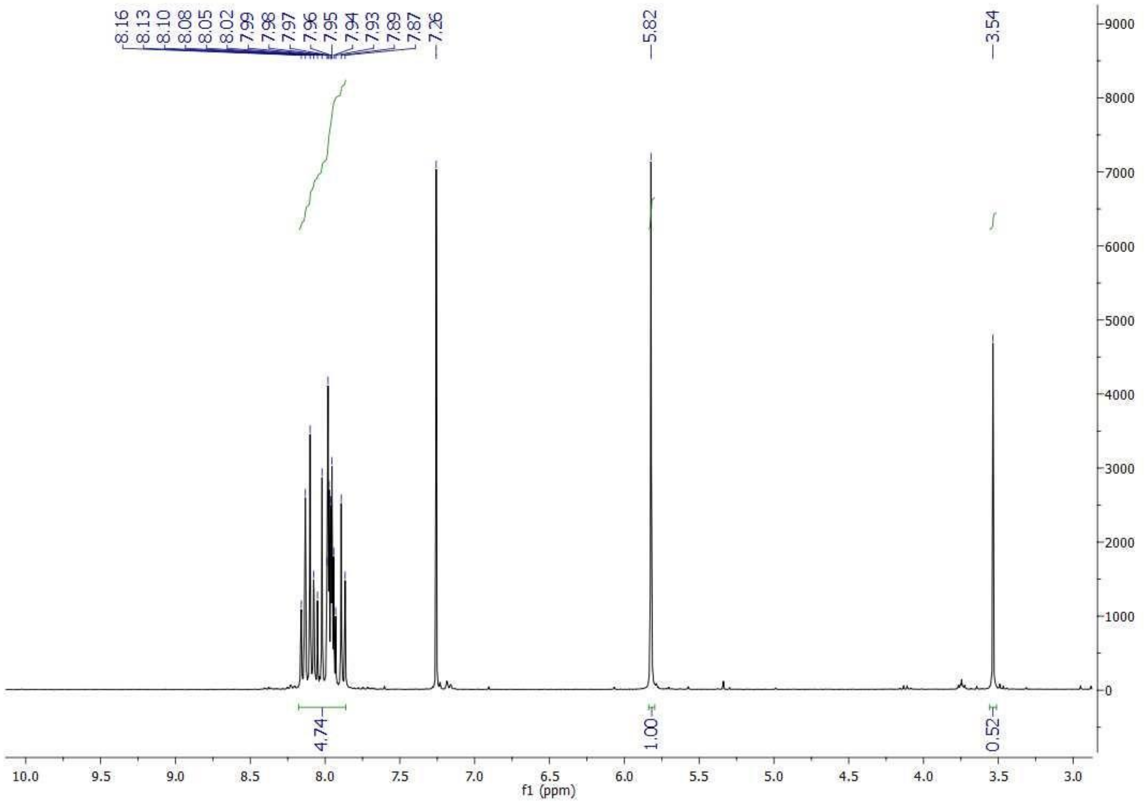

Figure S10. ^1H NMR spectrum of bis(pyren-1-ylmethyl) malonate (BP).

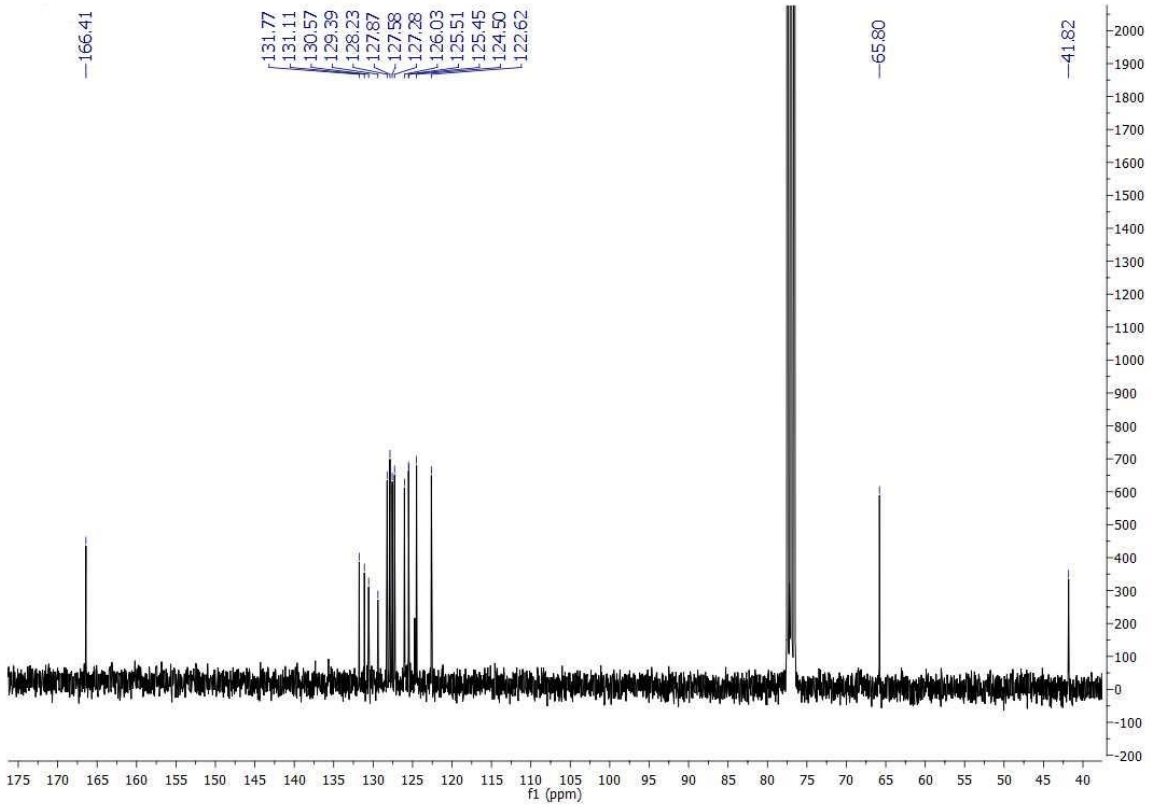

Figure S11. ^{13}C NMR spectrum of bis(pyren-1-ylmethyl) malonate (BP).

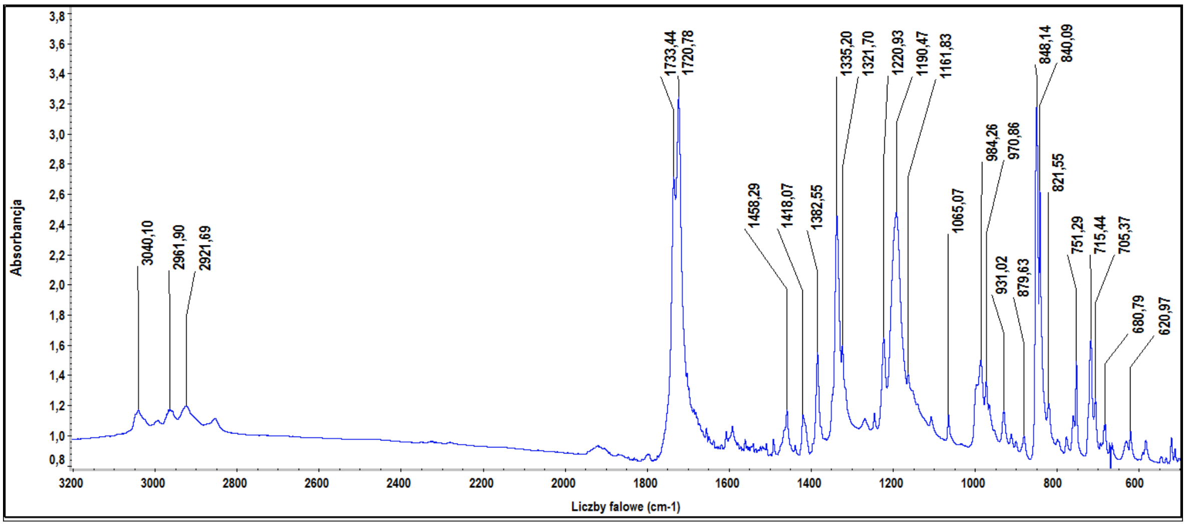

Figure S12. FT-IR spectrum of bis(pyren-1-ylmethyl) malonate (BP).

Synthesis of methanofullerenes

61-(Ethyloxycarbonyl)-61-(pyren-1-yl methyloxycarbonyl)-1,2-methano[60]fullerene ([C60]B1)

(reported earlier by Echegoyen et al.)[2]

Yield: 49%; ESI-MS (registered after reduction with sodium-potassium alloy):[3] 1064.8 $[M]^-$; FT-IR (KBr) ν_{max} (cm^{-1}) 3040.76, 2974.80, 1742.96, 1537.17, 1459.08, 1427.20, 1364.08, 1288.71, 1265.57, 1232.35, 1203.47, 1187.25, 1174.74, 1112.40, 1094.09, 1052.21, 1008.01, 841.51, 755.79, 704.40, 579.99, 554.97, 526.71; ^{1}H NMR (500 MHz, $CDCl_3$) δ 8.49-8.03 (m, 9H), 6.30 (s, 2H), 4.41 (ABq, 2H, $\Delta\delta_{AB}$ = 12.39Hz, J_{AB} = 7.16 Hz), 1.26 (t, J = 7.2Hz, 3H); ^{13}C NMR (125MHz, $CDCl_3$) δ 163.54, 163.01, 145.15, 145.04, 144.98, 144.78, 144.73, 144.52, 144.09, 144.01, 143.78, 143.55, 142.95, 142.85, 142.78, 142.65, 142.09, 142.06, 141.73, 141.36, 140.77, 140.74, 140.52, 139.61, 137.90, 132.44, 131.17, 130.72, 130.24, 129.23, 128.57, 128.24, 127.72, 127.33, 126.25, 125.77, 125.66, 125.01, 124.70, 124.59, 123.21, 71.40, 67.55, 63.48, 13.97 ppm;

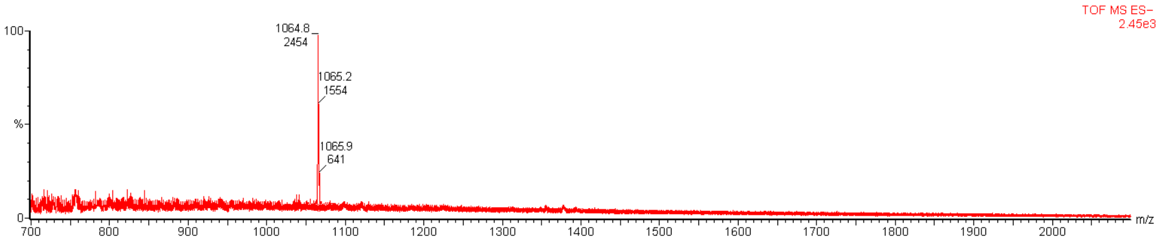

Figure S13. ESI-MS spectrum of 61-(ethyloxycarbonyl)-61-(pyren-1-yl methyloxycarbonyl)-1,2-methano[60]fullerene (**[C60]B1)**.

---

[2] M. Ángeles Herranz, Charles T. Cox, Luis Echegoyen, Retrocyclopropanation Reactions of Fullerenes: Complete Product Analyses, *Journal of Organic Chemistry*, **2003**, *68*, 5009 – 5012.
[3] K. Hiraoka, I. Kudaka, S. Fujimaki, H. Shinohara, *Rap. Comm. Mass Spectrom.*, **1992**, *6*, 254–256.

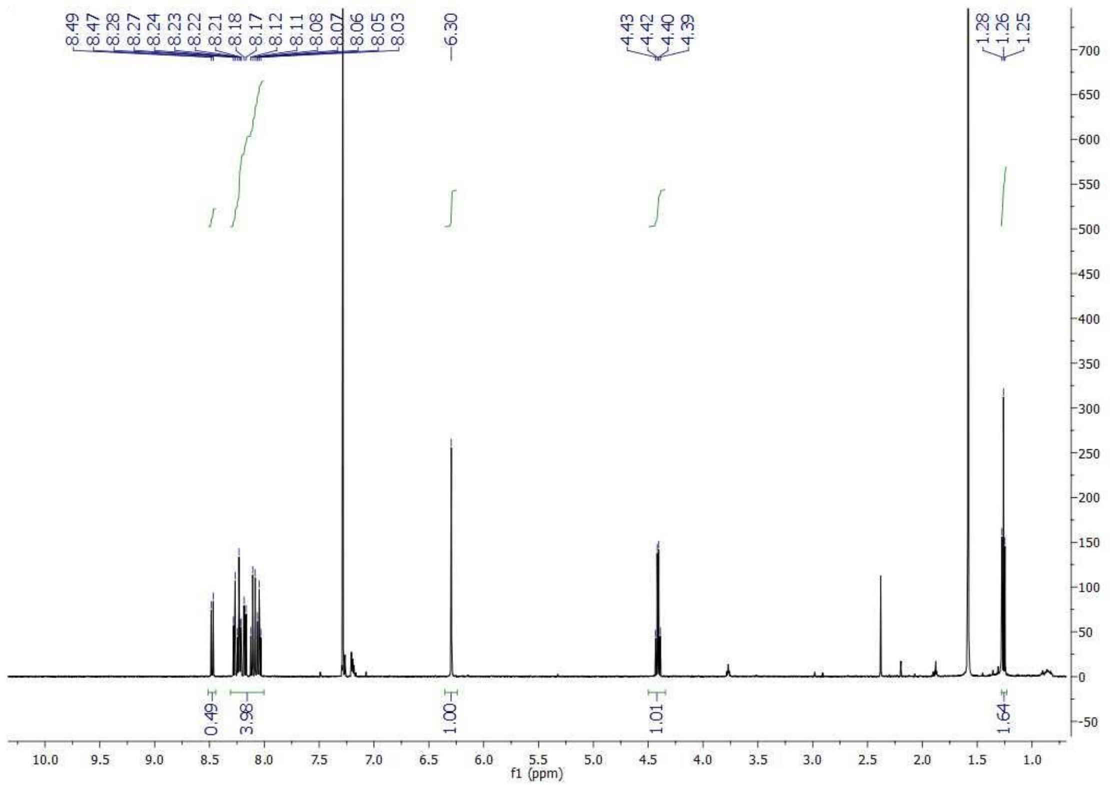

Figure S14. ^{1}H NMR spectrum of 61-(ethyloxycarbonyl)-61-(pyren-1-yl methyloxycarbonyl)-1,2-methano[60]fullerene (**[C60]B1)**.

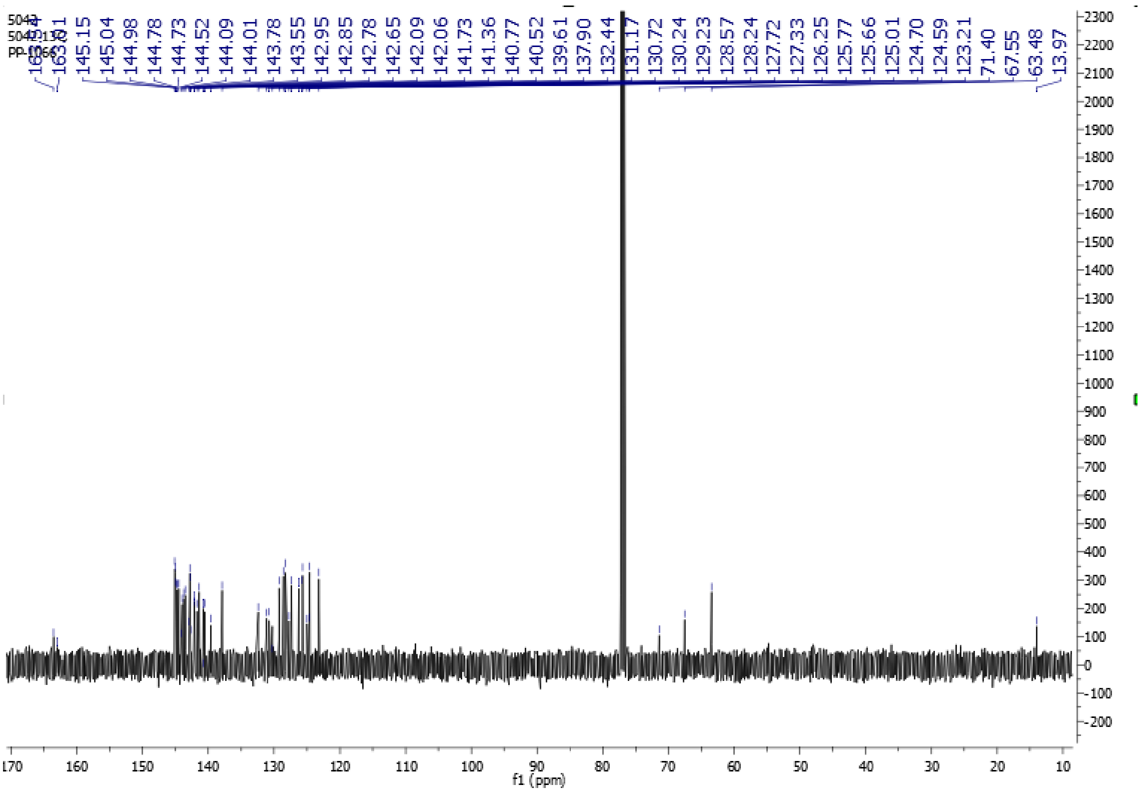

Figure S15. ^{13}C NMR spectrum of 61-(ethyloxycarbonyl)-61-(pyren-1-yl methyloxycarbonyl)-1,2-methano[60]fullerene (**[C60]B1)**.

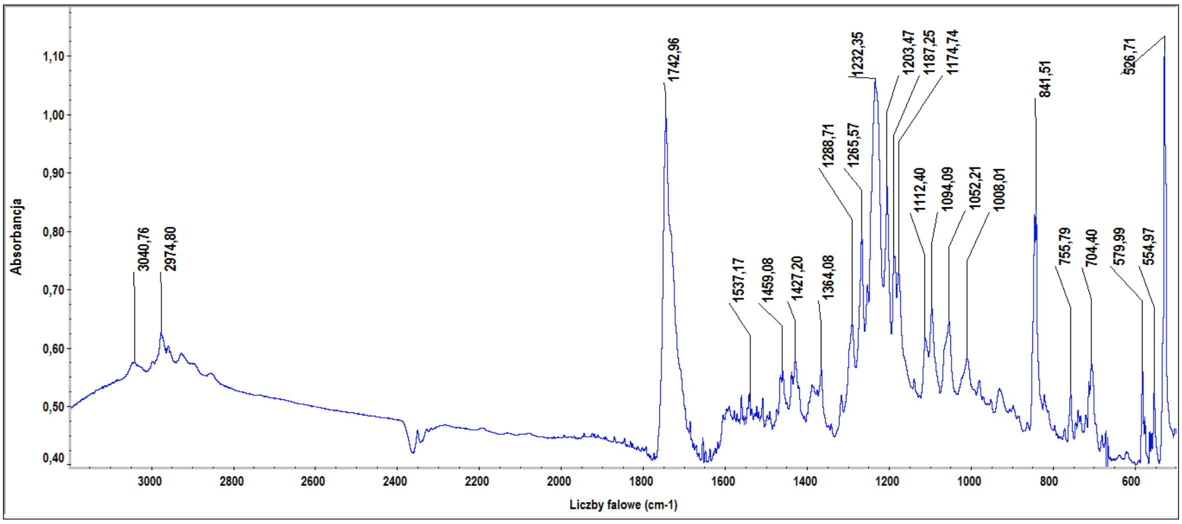

Figure S16. FT-IR spectrum of 61-(ethyloxycarbonyl)-61-(pyren-1-yl methyloxycarbonyl)-1,2-methano[60]fullerene (**[C60]B1)**.

61-Bis(pyren-1-yl methyloxycarbonyl)-1,2-methano[60]-fullerene ([C60]B2) (reported earlier by Echegoyen et al.)[4]

Yield: 27%; ESI-MS (registered after reduction with sodium-potassium alloy): 1251.0 $[M]^-$ FT-IR (KBr) ν_{max} (cm^{-1}) 3044.24, 2964.09, 1757.90, 1734.56, 1512.52, 1428.34, 1372.41, 1266.34, 1257.75, 1232.19, 1203.40, 1184.76, 1111.21, 1064.0, 1002.10, 964.68, 926.27, 845.98, 756.07, 705.18; ^{1}H NMR (500 MHz, $CDCl_3$) δ 8.22–7.89 (m, 9H), 6.03 (s, 2H) ppm; ^{13}C NMR was not registered due to low solubility.

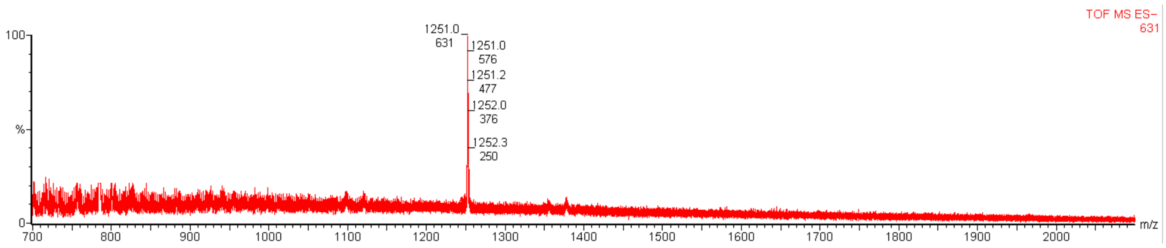

Figure S17. ESI-MS spectrum of 61-bis(pyren-1-yl methyloxycarbonyl)-1,2-methano[60]-fullerene (**[C60]B2)**.

[4] M. Ángeles Herranz, Charles T. Cox, Luis Echegoyen, Retrocyclopropanation Reactions of Fullerenes: Complete Product Analyses, *Journal of Organic Chemistry*, **2003**, *68*, 5009 – 5012.

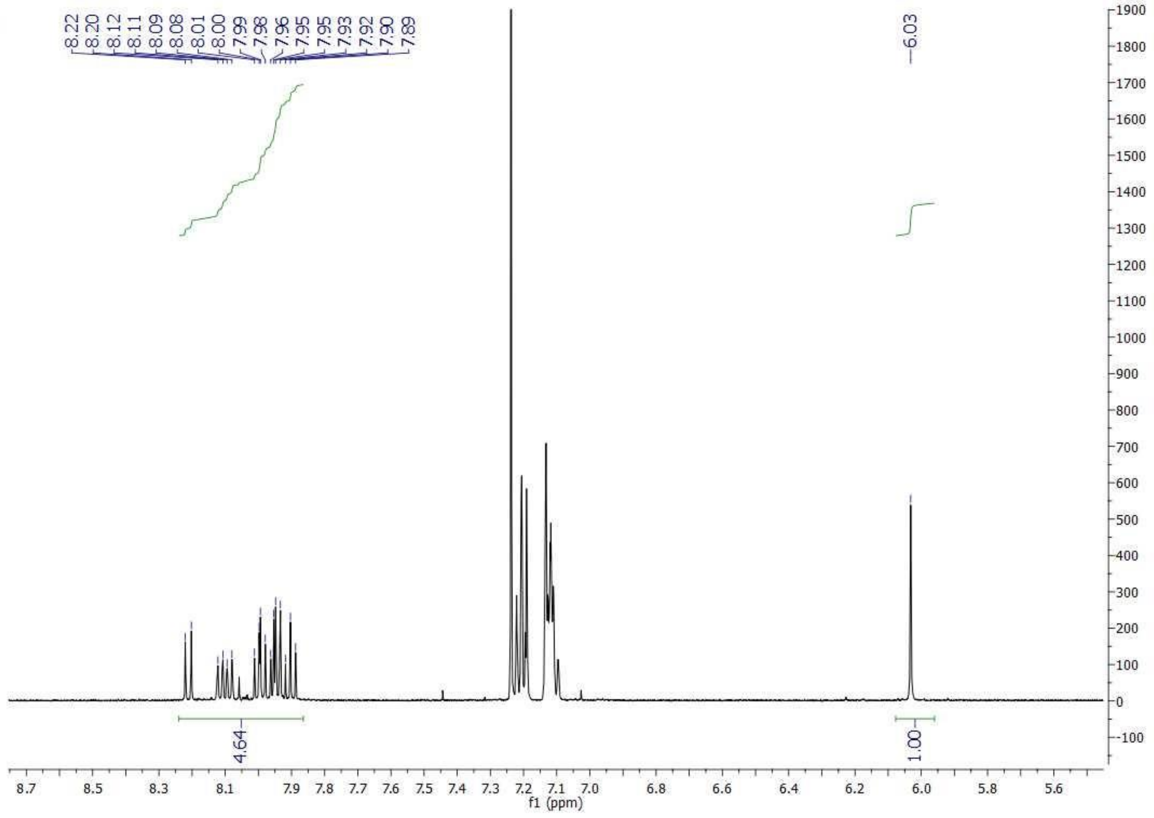

Figure S18. ^{1}H NMR spectrum of 61-bis(pyren-1-yl methyloxycarbonyl)-1,2-methano[60]-fullerene (**[C60]B2**).

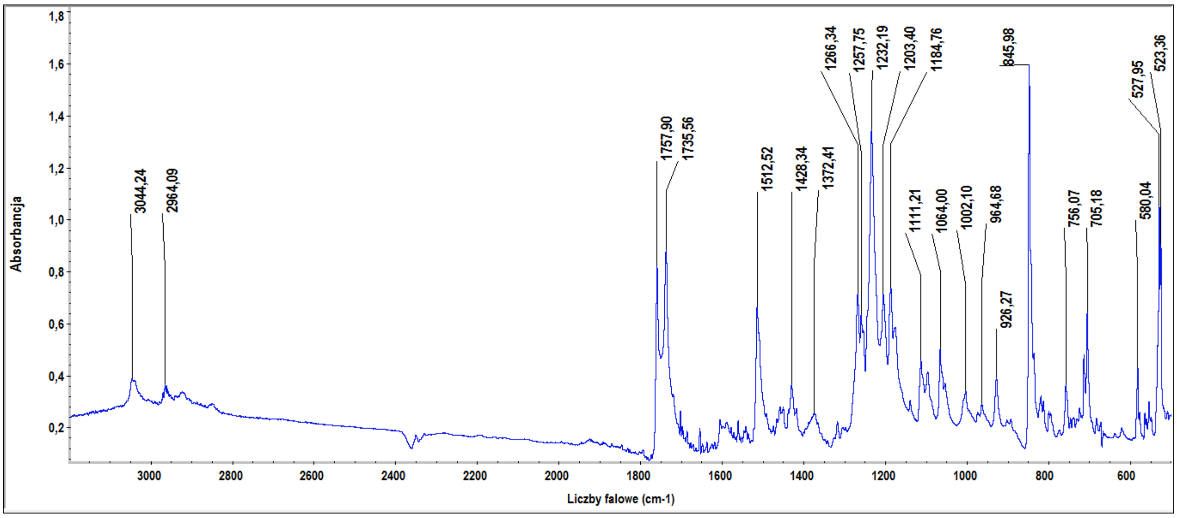

Figure S19. FT-IR spectrum of 61-bis(pyren-1-yl methyloxycarbonyl)-1,2-methano[60]-fullerene (**[C60]B2**).

71-(Ethyloxycarbonyl)-71-(pyren-1-yl methyloxycarbonyl)-1,2-methano[70]fullerene ([C70]B1)

Yield: 62%; ESI-MS (registered after reduction with sodium-potassium alloy): 1185.3 $[M]^{-}$; FT-IR (KBr) ν_{max} (cm^{-1}) 3040.76, 2970.25, 2922.58, 1743.54, 1455.14, 1428.21, 1415.59, 1364.09, 1267.97, 1226.28, 1214.79, 1175.99, 1159.69, 1135.89, 1091.93, 1083.87, 1036.18, 1004.07, 952.86, 841.51, 794.87, 755.37, 725.75, 704.74, 672.71, 577.97, 534.28; ^{1}H NMR (500 MHz, $CDCl_3$) δ 8.47-7.96 (m, 9H), 6.22 (ABq, 2H, $\Delta\delta_{AB}$ = 39.58Hz, J_{AB} = 11.90Hz), 4.38 (q, J = 8.3Hz, 2H), 1.28 (t, J = 7.1Hz, 3H) ppm; ^{13}C NMR (125 MHz, $CDCl_3$) δ 163.52, 163.12, 155.28, 154.51, 151.22, 151.14, 151.10, 151.04, 150.99, 150.95, 150.55, 150.47, 150.39, 149.17, 149.05, 148.99, 148.76, 148.58, 148.43, 148.38, 148.36, 148.34, 148.16, 147.64, 147.45, 147.38, 147.18, 147.13, 147.09, 146.91, 146.64, 146.35, 145.84, 145.40, 145.30, 144.72, 144.14, 143.83, 143.81, 143.71, 143.56, 143.35, 142.78, 142.56, 142.25, 142.20, 141.51, 141.30, 140.99, 140.72, 139.18, 136.73, 136.44, 133.34, 132.89, 132.70, 132.49, 132.41, 131.14, 130.85, 130.80, 130.74, 130.67, 130.38, 129.92, 129.46, 129.05, 128.71, 128.21, 127.63, 127.27, 126.29, 125.79, 125.67, 125.07, 124.77, 124.59, 123.39, 67.59, 63.61, 29.72, 14.03 ppm.

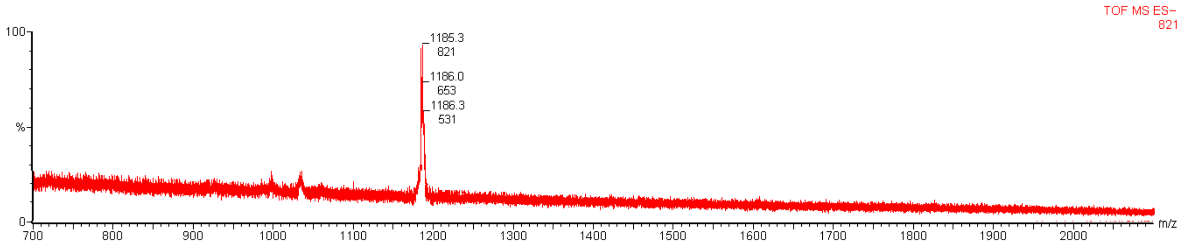

Figure S20. ESI-MS spectrum of 71-(ethyloxycarbonyl)-71-(pyren-1-yl methyloxycarbonyl)-1,2-methano[70]fullerene (**[C70]B1**).

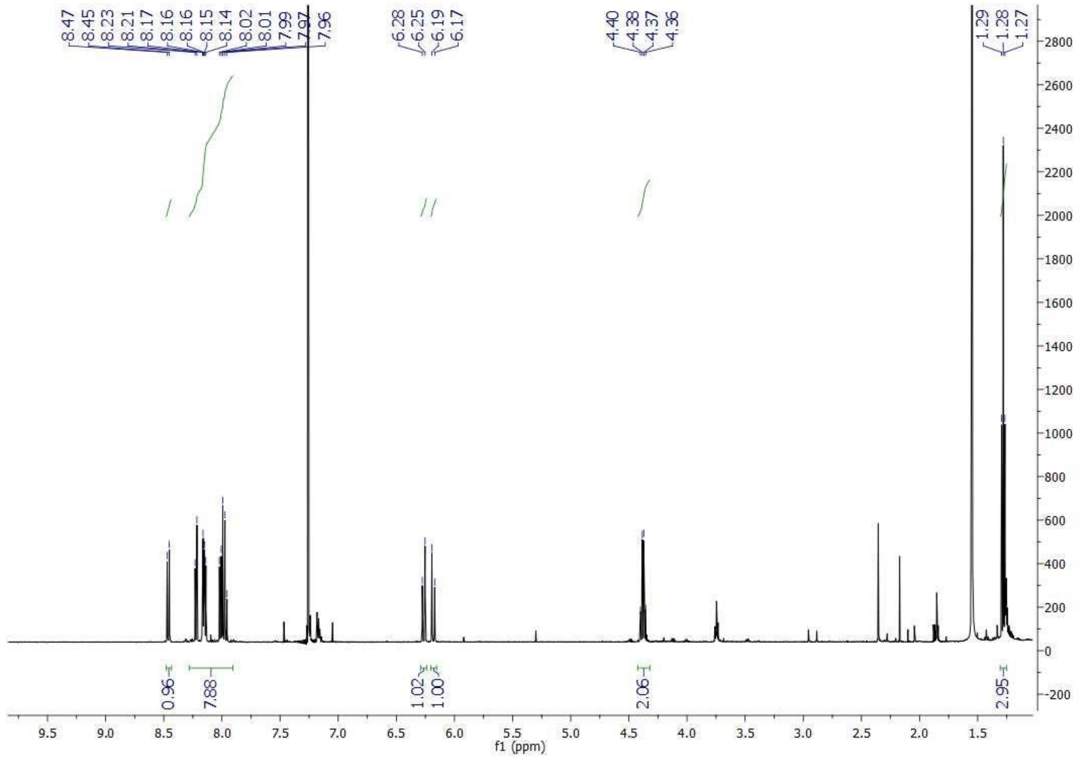

Figure S21. ^{1}H NMR spectrum of 71-(ethyloxycarbonyl)-71-(pyren-1-yl methyloxycarbonyl)-1,2-methano[70]fullerene (**[C70]B1)**.

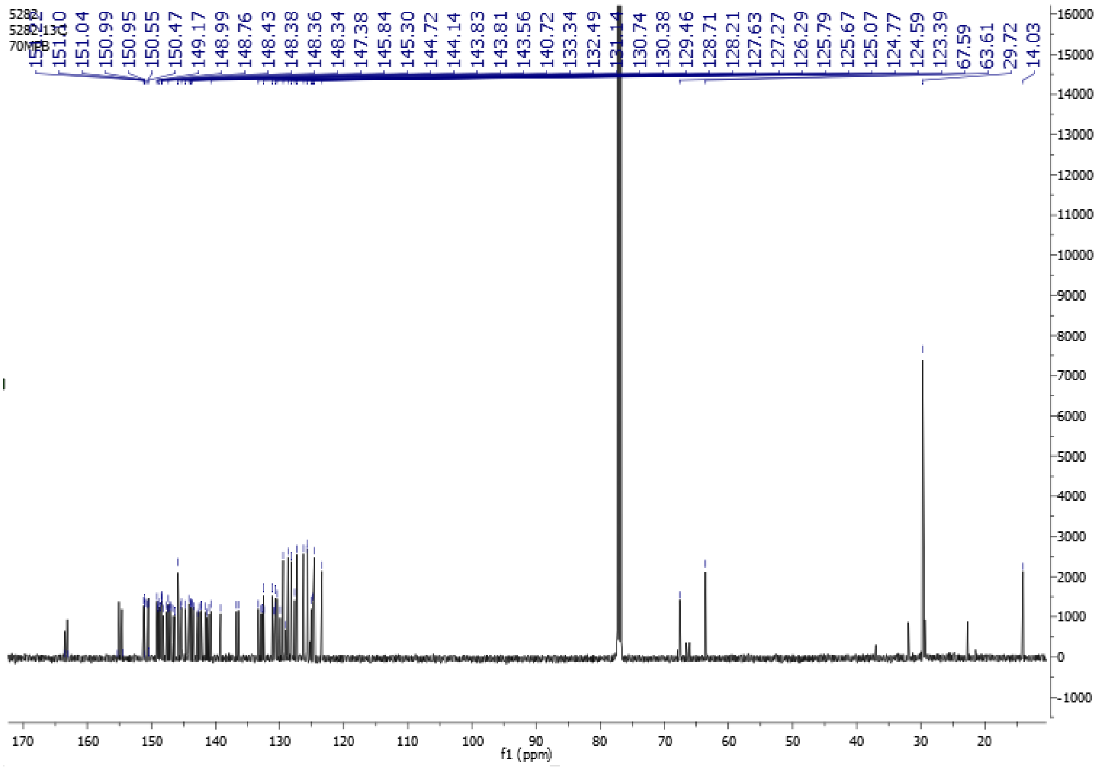

Figure S22. ^{13}C NMR spectrum of 71-(ethyloxycarbonyl)-71-(pyren-1-yl methyloxycarbonyl)-1,2-methano[70]fullerene (**[C70]B1)**.

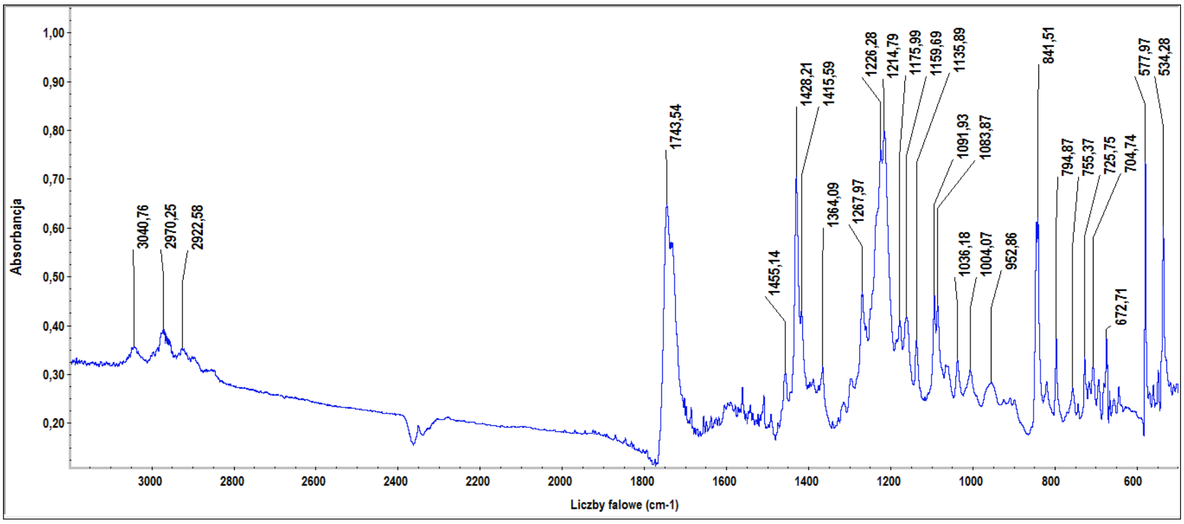

Figure S23. FT-IR spectrum of 71-(ethyloxycarbonyl)-71-(pyren-1-yl methyloxycarbonyl)-1,2-methano[70]fullerene (**[C70]B1)**.

71-Bis(pyren-1-yl methyloxycarbonyl)-1,2-methano[70]-fullerene ([C70]B2)

Yield: 33%; ESI-MS (registered after reduction with sodium-potassium alloy): 1371.9 $[M]^{-}$; FT-IR (KBr) ν_{max} (cm^{-1}) 3040.95, 2924.55, 2853.64, 1747.74, 1559.35, 1513.83, 1507.04, 1454.31, 1428.21, 1361.64, 1258.99, 1212.38, 1157.61, 1078.17, 1028.15, 846.08, 796.16, 752.13, 704.53, 677.10, 578.88, 534.00; ^{1}H NMR (500 MHz, $CDCl_3$) δ 8.30-9.93 (m, 9H), 6.05 (ABq, 2H, $\Delta\delta_{AB}$ = 27.90Hz, J_{AB} = 11.98Hz) ppm; ^{13}C NMR was not registered due to low solubility.

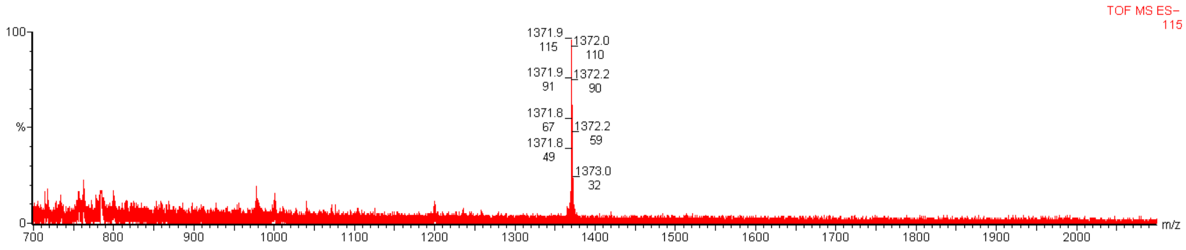

Figure S24. ESI-MS spectrum of 71-bis(pyren-1-yl methyloxycarbonyl)-1,2-methano[70]-fullerene (**[C70]B2)**.

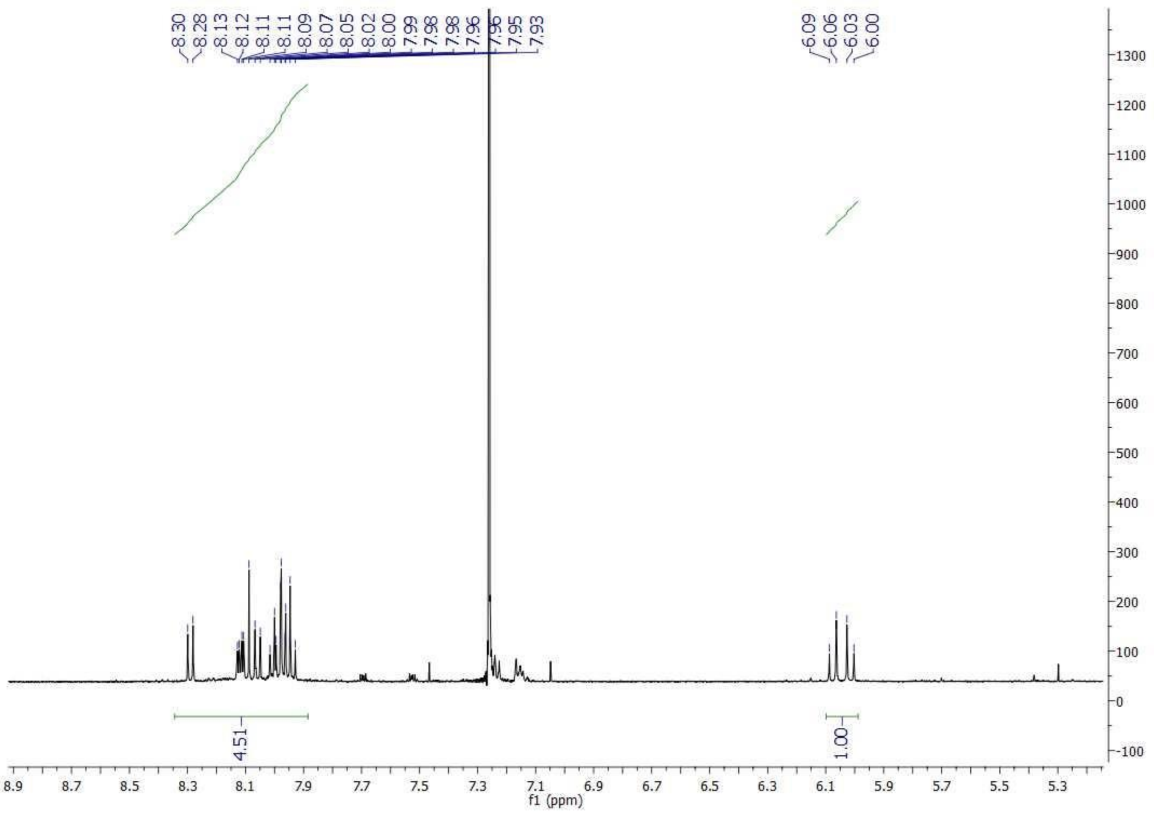

Figure S25. ^{1}H NMR spectrum of 71-bis(pyren-1-yl methyloxycarbonyl)-1,2-methano[70]-fullerene (**[C70]B1**).

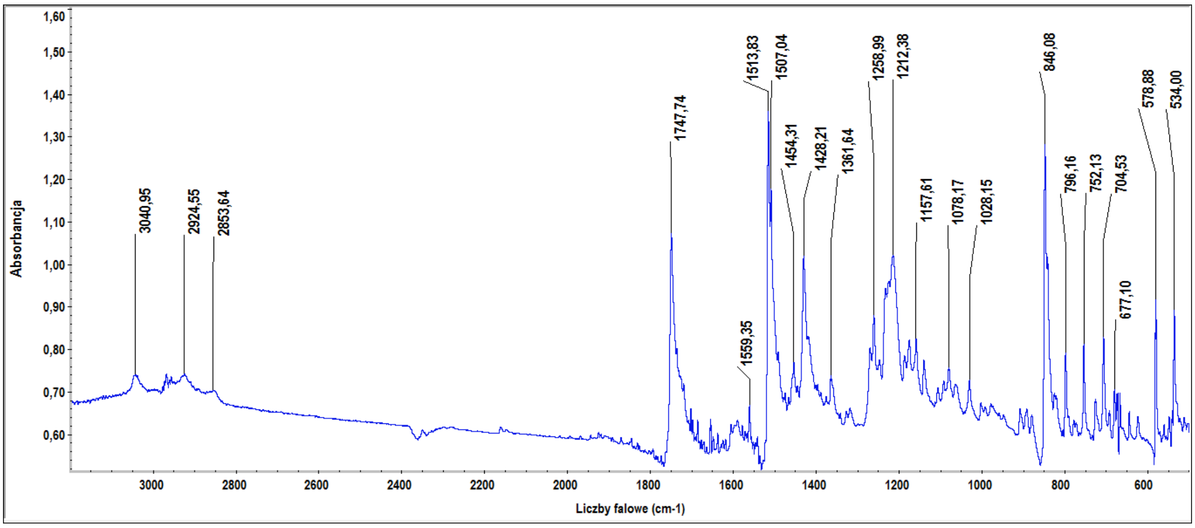

Figure S26. FT-IR spectrum of 71-bis(pyren-1-yl methyloxycarbonyl)-1,2-methano[70]-fullerene (**[C70]B1**).

Synthesis of fulleropyrrolidines

N-methyl-2-[pyren-1-yl]-3,4-[60]fulleropyrrolidine ([C60]P1)

Yield: 49%, ESI-MS: 978.7 $[M+H]^+$; FT-IR (KBr) $\nu_{max}(cm^{-1})$ 3036.82, 2937.61, 2831.97, 2773.86, 1512.64, 1505.22, 1461.66, 1427.68, 1332.52, 1240.63, 1179.24, 1031.02, 849.14, 837.49, 821.41, 706.32, 594.58, 574.48, 526.04; ^{1}H NMR (500 MHz, $CDCl_3$) δ 8.89-7.94 (m, 9H), 6.15 (s, 1H), 4.83 (AXq, 2H,$\Delta\delta_{AB}$=311.66Hz, J_{AX}=9.38Hz), 2.85 (s, 3H) ppm; ^{13}C NMR was not registered due to low solubility.

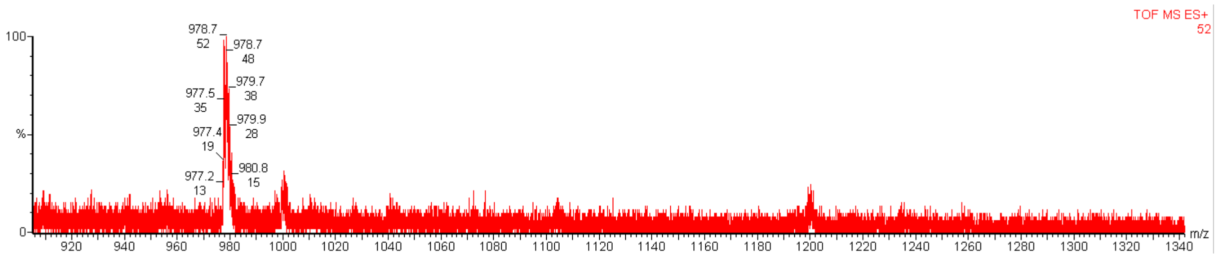

Figure S27. ESI-MS spectrum of N-methyl-2-[pyren-1-yl]-3,4-[60]fulleropyrrolidine (**[C60]P1**).

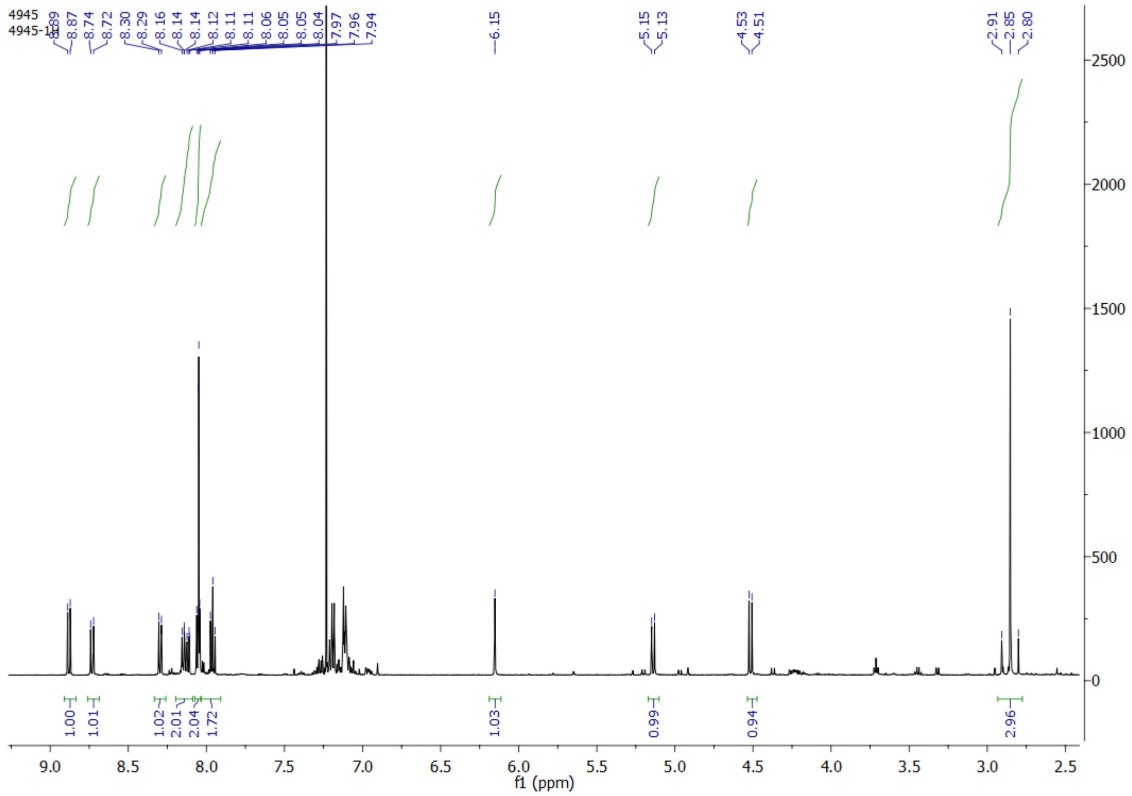

Figure S28. ^{1}H NMR spectrum of N-methyl-2-[pyren-1-yl]-3,4-[60]fulleropyrrolidine (**[C60]P1**).

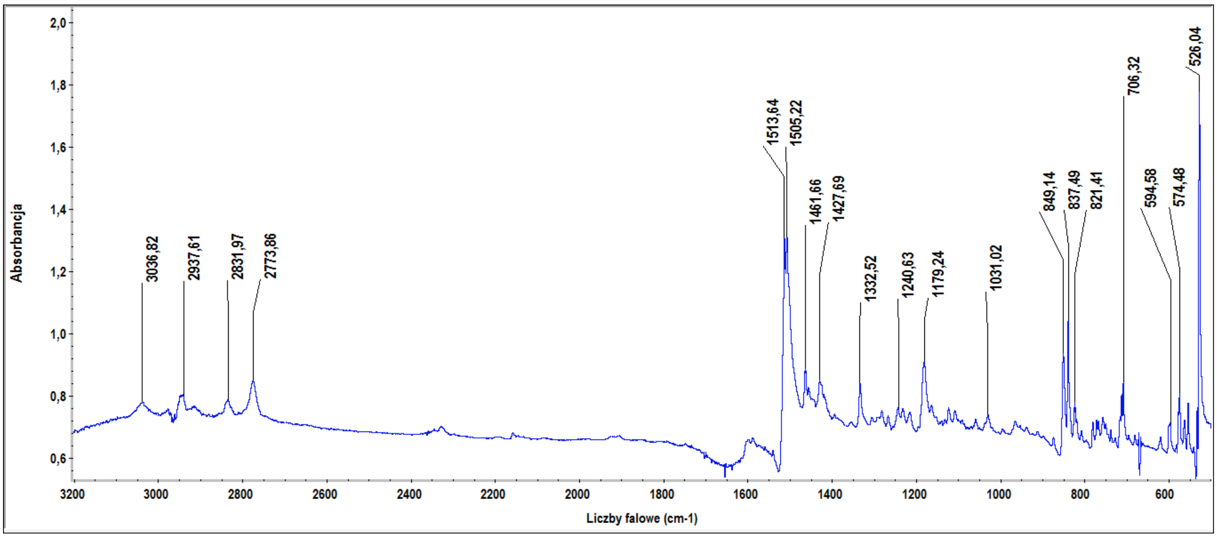

Figure S29. FT-IR spectrum of N-methyl-2-[pyren-1-yl]-3,4-[60]fulleropyrrolidine (**[C60]P1**).

N-methyl-2-[pyren-1-yl]-3,4-[70]fulleropyrrolidine ([C70]P1)

Yield: 28%, ESI-MS: 1099.2 $[M+H]^+$; FT-IR (KBr) $\nu_{max}(cm^{-1})$ 3038.79, 2957.71, 2923.25, 2847.49, 2777.65, 1624.34, 1428.52, 1413.55, 1330.74, 1271.68, 1020.14, 848.02, 836.25, 795.00, 726.34, 713.42, 673.81, 641.41, 577.64, 534.48; ^{1}H NMR (500 MHz, $CDCl_3$) δ 8.96-7.45 (pyrene rings), 5.45-3.43 (pyrrolidine hydrogens), 2.58-2.40 (*N*-methyl groups singlets) ppm; ^{13}C NMR spectrum was not analyzed in detail due to the mixture isomers obtained during the synthesis;

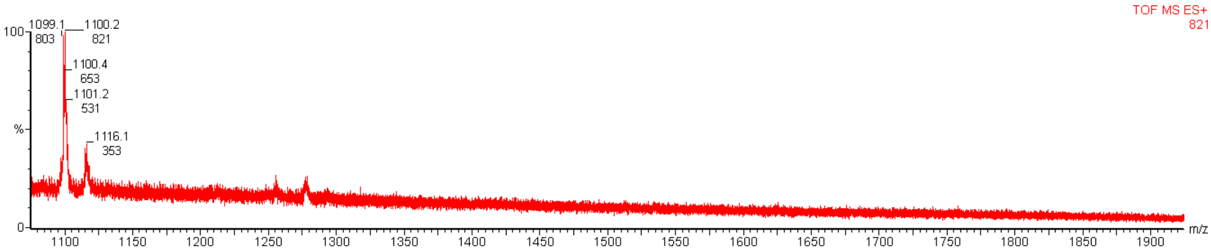

Figure S30. ESI-MS spectrum of *N*-methyl-2-[pyren-1-yl]-3,4-[70]fulleropyrrolidine (**[C70]P1**).

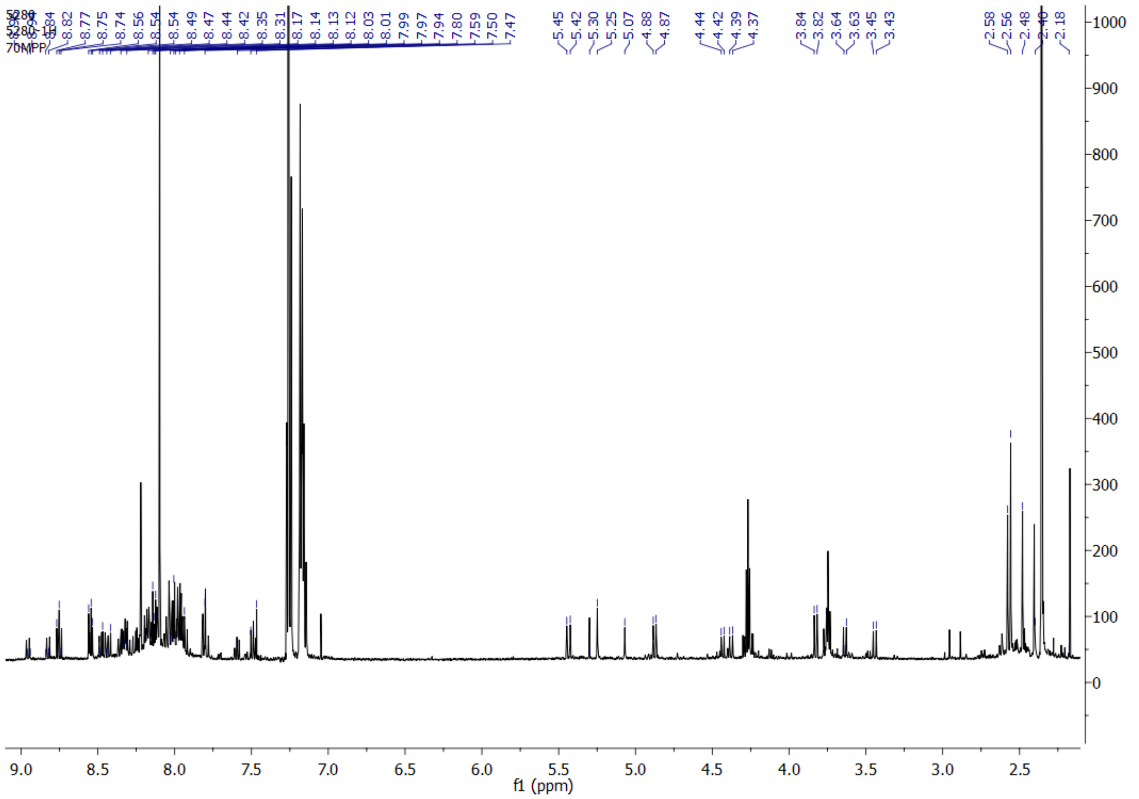

Figure S31. ^{1}H NMR spectrum of *N*-methyl-2-[pyren-1-yl]-3,4-[70]fulleropyrrolidine (**[C70]P1**).

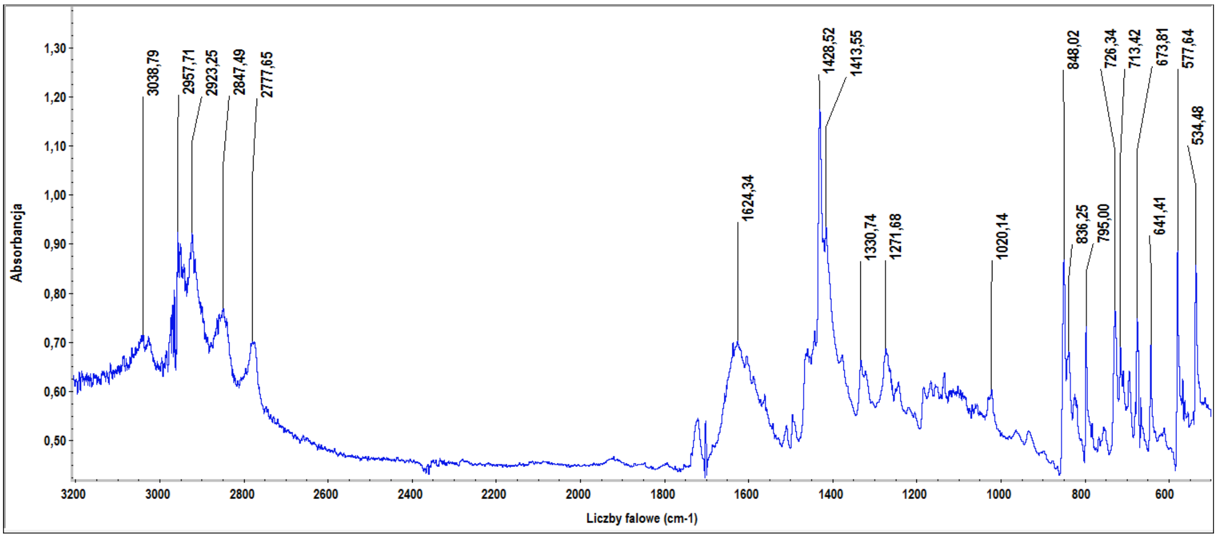

Figure S32. FT-IR spectrum of *N*-methyl-2-[pyren-1-yl]-3,4-[70]fulleropyrrolidine (**[C70]P1**).

Table S1. Optimized Geometries at DFT B3LYP/6-31g(d) level

[C60]P1

C	8.153160000000	-0.922706000000	1.874437000000
C	7.394450000000	0.012029000000	2.506843000000
C	7.822182000000	-1.373274000000	0.551326000000
C	6.674395000000	-0.820943000000	-0.098825000000
C	5.873823000000	0.164583000000	0.560192000000
C	6.334738000000	-1.264979000000	-1.411568000000
C	8.591339000000	-2.338051000000	-0.118221000000
C	8.249838000000	-2.762028000000	-1.401406000000
H	9.463285000000	-2.755093000000	0.379734000000
C	6.237184000000	0.584333000000	1.876756000000
C	5.450122000000	1.551291000000	2.512806000000
C	4.722690000000	0.723899000000	-0.091016000000
C	4.347640000000	2.097106000000	1.868454000000
H	5.714219000000	1.882357000000	3.514092000000
H	7.651288000000	0.346898000000	3.508872000000
C	5.174511000000	-0.702107000000	-2.034968000000
C	7.133230000000	-2.232633000000	-2.043243000000
H	8.857687000000	-3.510344000000	-1.902536000000
H	9.027012000000	-1.347617000000	2.362300000000
C	3.951838000000	1.710423000000	0.579758000000
C	4.413581000000	0.237531000000	-1.408373000000
H	4.902457000000	-1.045824000000	-3.030114000000
H	6.867371000000	-2.566293000000	-3.043282000000
C	2.713080000000	2.449786000000	0.035418000000
H	3.540318000000	0.617629000000	-1.916545000000
H	3.768121000000	2.859026000000	2.384706000000
C	1.353686000000	1.620521000000	-0.046348000000
N	2.790528000000	3.134983000000	-1.245761000000
H	2.540310000000	3.225263000000	0.793618000000
C	0.380604000000	2.175621000000	1.004166000000
C	1.436724000000	0.160571000000	0.430652000000
C	0.840442000000	1.786757000000	-1.563808000000
C	1.990457000000	2.551932000000	-2.288718000000
C	4.006172000000	3.821176000000	-1.622961000000

C	-0.682491000000	3.003090000000	0.723596000000
C	0.317855000000	1.302274000000	2.142285000000
C	0.961003000000	0.052080000000	1.785477000000
C	1.320075000000	-0.933264000000	-0.399529000000
C	-0.514850000000	2.501704000000	-1.696356000000
C	0.509096000000	0.468256000000	-2.274806000000
H	1.584182000000	3.337804000000	-2.940163000000
H	2.543401000000	1.861874000000	-2.953557000000
H	3.781105000000	4.549133000000	-2.411754000000
H	4.403009000000	4.370332000000	-0.762741000000
H	4.799855000000	3.149108000000	-1.992782000000
C	-1.848834000000	3.002986000000	1.578977000000
C	-1.148409000000	3.165951000000	-0.670336000000
C	-0.789068000000	1.308003000000	2.998682000000
C	0.463920000000	-1.152997000000	2.294763000000
C	0.851428000000	-0.775692000000	-1.794240000000
C	0.786076000000	-2.183087000000	0.096790000000
C	-1.310226000000	1.868959000000	-2.715057000000
C	-0.675651000000	0.614440000000	-3.074433000000
C	-3.025768000000	3.160899000000	0.744059000000
C	-1.901879000000	2.179581000000	2.709169000000
C	-2.592265000000	3.260214000000	-0.637980000000
C	-1.312851000000	0.053314000000	3.512958000000
C	-0.700313000000	-1.150285000000	3.166498000000
C	0.375396000000	-2.302576000000	1.428002000000
C	0.042623000000	-1.929768000000	-2.120098000000
C	0.000930000000	-2.798142000000	-0.958111000000
C	-2.706425000000	1.970663000000	-2.718953000000
C	-1.457314000000	-0.493150000000	-3.422523000000
C	-4.207691000000	2.492017000000	1.063849000000
C	-3.125427000000	1.470902000000	3.034881000000
C	-3.365024000000	2.684644000000	-1.651979000000
C	-2.761770000000	0.153958000000	3.533403000000
C	-1.510665000000	-2.307879000000	2.829270000000
C	-0.843963000000	-3.020763000000	1.751170000000
C	-1.088813000000	-1.798993000000	-2.932810000000
C	-1.163032000000	-3.502144000000	-0.649299000000

C	-3.517979000000	0.813289000000	-3.061686000000
C	-2.907431000000	-0.391772000000	-3.406981000000
C	-4.256074000000	1.623023000000	2.229050000000
C	-5.000710000000	1.879593000000	0.009754000000
C	-4.587093000000	1.974943000000	-1.320808000000
C	-3.542326000000	-0.956174000000	3.205178000000
C	-2.903865000000	-2.212362000000	2.846131000000
C	-1.599199000000	-3.608040000000	0.733766000000
C	-2.306261000000	-2.518018000000	-2.605321000000
C	-2.343708000000	-3.351739000000	-1.485340000000
C	-4.683217000000	0.816161000000	-2.194338000000
C	-3.433567000000	-1.647002000000	-2.899055000000
C	-5.067549000000	0.470151000000	1.891269000000
C	-5.528156000000	0.628769000000	0.519259000000
C	-4.717713000000	-0.795021000000	2.368133000000
C	-3.686523000000	-2.826661000000	1.788289000000
C	-3.045575000000	-3.511147000000	0.753384000000
C	-3.505843000000	-3.352402000000	-0.618741000000
C	-5.188661000000	-0.388976000000	-1.702454000000
C	-4.550713000000	-1.645540000000	-2.061600000000
C	-5.619755000000	-0.484555000000	-0.318908000000
C	-4.808323000000	-1.951184000000	1.492893000000
C	-4.588016000000	-2.515560000000	-0.899660000000
C	-5.249524000000	-1.799000000000	0.177532000000

}

[C70]P1

C	-1.503760000000	1.238116000000	3.195671000000
C	-2.580624000000	0.401555000000	3.490985000000
C	-1.584279000000	-0.810148000000	-3.388667000000
C	-0.202674000000	0.673562000000	2.886640000000
C	-0.028473000000	-0.714490000000	2.890266000000
C	0.757163000000	-1.326100000000	1.846739000000
C	-1.150630000000	-1.587398000000	3.181720000000
C	0.420194000000	1.491773000000	1.868902000000
C	1.188368000000	0.913236000000	0.882712000000
C	-0.519906000000	2.587353000000	1.542685000000
C	-2.402129000000	-1.041542000000	3.475120000000

C	-3.602155000000	-1.633281000000	2.916916000000
C	-1.052192000000	-2.749137000000	2.312289000000
C	-3.508311000000	-2.747740000000	2.080469000000
C	-4.527153000000	-0.556498000000	2.593141000000
C	-3.898018000000	0.699513000000	2.952026000000
C	-4.087744000000	1.830509000000	2.155114000000
C	-5.320575000000	-0.638673000000	1.446876000000
C	-4.914272000000	1.745558000000	0.961435000000
C	-2.968673000000	2.707458000000	1.862632000000
C	0.131162000000	-2.587920000000	1.485175000000
C	1.326235000000	-0.518595000000	0.856218000000
C	1.360645000000	1.485261000000	-0.531667000000
C	-1.697142000000	2.413387000000	2.366148000000
C	-0.640263000000	3.032144000000	0.246086000000
C	-3.098543000000	3.159756000000	0.499280000000
C	0.115003000000	-3.003284000000	0.154439000000
C	1.309844000000	-0.952701000000	-0.527407000000
C	-2.208067000000	-3.316319000000	1.771804000000
C	0.724495000000	-2.174203000000	-0.872472000000
C	-1.085536000000	-3.595603000000	-0.408784000000
C	1.159607000000	0.210738000000	-1.359300000000
C	-2.225198000000	-3.748590000000	0.383424000000
C	0.329691000000	2.672570000000	-0.886406000000
C	2.774232000000	2.163658000000	-0.788386000000
C	-1.949436000000	3.292670000000	-0.289698000000
C	0.365870000000	0.137709000000	-2.485006000000
C	-0.669813000000	2.338911000000	-1.998478000000
C	1.273608000000	3.851217000000	-1.256348000000
N	2.507562000000	3.583475000000	-0.537358000000
C	3.924417000000	1.610922000000	0.034421000000
H	2.991399000000	2.022132000000	-1.861688000000
C	-1.967905000000	2.862364000000	-1.675621000000
C	-4.329553000000	-2.828035000000	0.884582000000
C	-5.216666000000	-1.796122000000	0.574435000000
C	-3.535731000000	-3.445743000000	-0.164292000000
C	-5.517413000000	0.534988000000	0.614399000000
C	-5.347866000000	-1.337144000000	-0.798050000000

C	-3.656449000000	-3.002195000000	-1.483017000000
C	-5.533632000000	0.102812000000	-0.773974000000
C	-4.301740000000	2.570744000000	-0.065283000000
C	-4.319102000000	2.157137000000	-1.396729000000
C	-4.581156000000	-1.925245000000	-1.806512000000
C	-4.947978000000	0.899132000000	-1.759939000000
C	-3.132536000000	2.314061000000	-2.222685000000
C	-0.088057000000	-2.254443000000	-2.061856000000
C	-1.211637000000	-3.131016000000	-1.781009000000
C	-0.574697000000	1.233212000000	-2.814581000000
C	-0.275972000000	-1.108840000000	-2.844412000000
H	0.854510000000	4.821273000000	-0.970694000000
H	1.431992000000	3.860668000000	-2.352847000000
C	3.587090000000	4.479253000000	-0.935676000000
C	4.107736000000	2.099277000000	1.335295000000
C	4.806415000000	0.619534000000	-0.465212000000
C	-2.472910000000	-2.839586000000	-2.304541000000
C	-2.661560000000	-1.658479000000	-3.131585000000
C	-1.769136000000	0.631685000000	-3.367319000000
H	3.294511000000	5.512304000000	-0.721079000000
H	4.491307000000	4.252404000000	-0.366167000000
H	3.824962000000	4.403566000000	-2.013864000000
C	5.138071000000	1.647027000000	2.148339000000
H	3.425756000000	2.861210000000	1.697653000000
C	5.874531000000	0.151294000000	0.372133000000
C	4.694895000000	0.043533000000	-1.782370000000
C	-3.031037000000	1.165828000000	-3.090083000000
C	-4.148896000000	0.283709000000	-2.808235000000
C	6.037712000000	0.673385000000	1.691301000000
H	5.254382000000	2.049954000000	3.151492000000
C	6.787382000000	-0.840695000000	-0.103406000000
C	5.565915000000	-0.901612000000	-2.231412000000
H	3.887199000000	0.354997000000	-2.434134000000
C	-3.968809000000	-1.098401000000	-2.827818000000
C	7.115285000000	0.190943000000	2.509394000000
C	7.851979000000	-1.302015000000	0.732230000000
C	6.644214000000	-1.380061000000	-1.416803000000

H	5.450237000000	-1.315447000000	-3.230326000000
C	7.982932000000	-0.752183000000	2.052418000000
H	7.222234000000	0.600321000000	3.510983000000
C	8.735661000000	-2.276129000000	0.239983000000
C	7.550590000000	-2.353623000000	-1.866217000000
H	8.794121000000	-1.108080000000	2.682918000000
C	8.585742000000	-2.794700000000	-1.044752000000
H	9.544345000000	-2.625175000000	0.877512000000
H	7.436079000000	-2.760597000000	-2.867924000000
H	9.279944000000	-3.547886000000	-1.407325000000

}

[C60]B1

C	0.649758000000	6.770120000000	0.839948000000
C	0.071808000000	2.252356000000	-0.762502000000
C	-0.359953000000	1.593634000000	-2.034310000000
C	0.011030000000	0.303818000000	-2.372791000000
C	0.747539000000	-0.548060000000	-1.420200000000
C	-0.902426000000	-0.543624000000	-3.106360000000
C	1.084541000000	-0.086404000000	-0.156283000000
C	0.267854000000	-1.906246000000	-1.589747000000
C	-2.169347000000	-0.076611000000	-3.471732000000
C	-0.745480000000	-1.906412000000	-2.630459000000
C	-1.163646000000	2.911569000000	-0.229211000000
C	-2.179470000000	2.860262000000	-1.252943000000
C	-1.686864000000	2.054362000000	-2.355064000000
C	-2.571885000000	1.253766000000	-3.084509000000
C	-3.537276000000	2.833386000000	-0.921914000000
C	-1.537434000000	2.843699000000	1.104849000000
C	-0.808849000000	1.985756000000	2.058969000000
C	-2.942734000000	2.793765000000	1.459815000000
C	-3.981976000000	1.219856000000	-2.733187000000
C	0.860546000000	1.329575000000	0.278482000000
C	0.882179000000	-0.944200000000	0.987053000000
C	0.272616000000	1.225698000000	1.648882000000
C	0.387674000000	-0.140489000000	2.089709000000
C	1.486710000000	2.568341000000	-0.328896000000
C	2.634560000000	2.372251000000	-1.305246000000

C	1.684107000000	3.794764000000	0.551851000000
O	3.793537000000	2.584711000000	-0.658406000000
O	2.525670000000	2.046217000000	-2.465309000000
O	1.779670000000	4.880106000000	-0.238329000000
O	1.739220000000	3.802098000000	1.758199000000
C	0.125924000000	-2.754239000000	-0.486719000000
C	-3.326863000000	-0.947308000000	-3.360918000000
C	-1.854194000000	-2.746791000000	-2.533004000000
C	0.440179000000	-2.261242000000	0.833316000000
C	-1.032644000000	-3.624508000000	-0.377956000000
C	-4.449840000000	-0.144681000000	-2.904263000000
C	-3.173203000000	-2.255611000000	-2.900304000000
C	-2.004180000000	-3.619891000000	-1.379777000000
C	-0.527942000000	-0.683804000000	2.996107000000
C	-0.519254000000	-2.822895000000	1.770385000000
C	-1.432399000000	-3.666474000000	1.019244000000
C	-4.455000000000	1.993587000000	-1.674617000000
C	-5.372371000000	-0.682104000000	-2.004650000000
C	-4.134099000000	-2.815569000000	-1.968553000000
C	-3.411526000000	-3.659535000000	-1.028775000000
C	-3.928052000000	2.799867000000	0.467651000000
C	-1.777041000000	1.428400000000	2.977619000000
C	-3.091891000000	1.925899000000	2.614795000000
C	-5.416521000000	1.437083000000	-0.739023000000
C	-5.090690000000	1.936636000000	0.586832000000
C	-5.865931000000	0.124924000000	-0.900207000000
C	-5.228976000000	1.103384000000	1.697732000000
C	-1.635932000000	0.120319000000	3.452684000000
C	-4.211141000000	1.102547000000	2.736239000000
C	-2.799004000000	-0.741993000000	3.569955000000
C	-4.061199000000	-0.261555000000	3.218404000000
C	-5.211949000000	-2.044664000000	-1.528204000000
C	-0.993446000000	-2.050509000000	2.829447000000
C	-2.401507000000	-2.086471000000	3.184595000000
C	5.040588000000	2.461296000000	-1.417903000000
C	1.978181000000	6.156077000000	0.428168000000
C	6.007441000000	1.574435000000	-0.682465000000

H	5.436697000000	3.476614000000	-1.498650000000
H	4.792742000000	2.099703000000	-2.416218000000
H	2.492190000000	6.767153000000	-0.317173000000
H	2.633250000000	6.001180000000	1.288559000000
C	7.046360000000	2.168790000000	0.040192000000
C	5.889347000000	0.162960000000	-0.704500000000
H	0.820955000000	7.760512000000	1.276940000000
H	-0.011257000000	6.884598000000	-0.024997000000
H	0.150360000000	6.146945000000	1.586941000000
C	-2.786726000000	-3.701117000000	1.357619000000
C	-3.795721000000	-3.697454000000	0.313052000000
C	-3.281165000000	-2.894821000000	2.462152000000
C	-6.010181000000	-0.739865000000	0.257711000000
C	-5.607458000000	-2.081504000000	-0.130042000000
C	-4.914649000000	-2.890732000000	0.771137000000
C	-4.595138000000	-2.393691000000	2.099116000000
C	-5.699434000000	-0.259112000000	1.531254000000
C	-4.977056000000	-1.103064000000	2.471183000000
C	7.977278000000	1.410013000000	0.740788000000
H	7.125961000000	3.253265000000	0.051768000000
C	4.848557000000	-0.524289000000	-1.422938000000
C	6.841519000000	-0.623939000000	0.018424000000
C	7.895411000000	0.010485000000	0.746773000000
H	8.775910000000	1.899872000000	1.291929000000
C	4.760386000000	-1.883857000000	-1.419613000000
H	4.114308000000	0.048692000000	-1.978606000000
C	6.746818000000	-2.049113000000	0.017113000000
C	8.838241000000	-0.805221000000	1.461664000000
C	5.699454000000	-2.697504000000	-0.703490000000
H	3.959820000000	-2.376520000000	-1.966091000000
C	7.697559000000	-2.834685000000	0.738945000000
C	8.744868000000	-2.162682000000	1.457437000000
H	9.634716000000	-0.308962000000	2.010717000000
C	5.622417000000	-4.100004000000	-0.690039000000
C	7.581339000000	-4.233462000000	0.722875000000
H	9.466086000000	-2.766121000000	2.003343000000
C	6.554669000000	-4.857302000000	0.015388000000

H	4.822606000000	-4.590745000000	-1.239096000000
H	8.305307000000	-4.830135000000	1.272460000000
H	6.480801000000	-5.941320000000	0.015515000000

}

[C70]B1

C	3.078659000000	6.720573000000	1.194981000000
C	2.190046000000	2.565913000000	-0.059754000000
C	3.137021000000	2.456379000000	-1.250862000000
C	2.623784000000	3.604766000000	0.954186000000
O	3.518081000000	4.441882000000	0.399315000000
C	4.012320000000	5.521767000000	1.237934000000
O	2.207537000000	3.689695000000	2.086904000000
O	4.263550000000	1.831865000000	-0.871295000000
C	5.277842000000	1.680867000000	-1.911398000000
C	6.506709000000	1.057235000000	-1.311808000000
H	5.494877000000	2.669483000000	-2.322763000000
H	4.843000000000	1.075805000000	-2.713093000000
O	2.911179000000	2.869864000000	-2.363550000000
H	4.991684000000	5.755056000000	0.814886000000
H	4.134756000000	5.142257000000	2.254758000000
C	0.700549000000	2.423191000000	-0.335588000000
C	0.105709000000	2.053953000000	-1.648976000000
C	0.361285000000	0.851544000000	-2.272639000000
C	-1.206479000000	2.630968000000	-1.739691000000
C	1.534880000000	1.285098000000	0.415181000000
C	1.610985000000	-0.023370000000	-0.304548000000
C	1.064992000000	0.934477000000	1.790642000000
C	-0.434327000000	3.013454000000	0.432231000000
C	-0.698009000000	2.686925000000	1.747639000000
C	-1.538623000000	3.211921000000	-0.472034000000
C	1.456780000000	-1.083703000000	0.657372000000
C	1.123898000000	-0.199888000000	-1.593391000000
C	1.119518000000	-0.498004000000	1.936805000000
C	0.057123000000	1.636056000000	2.433582000000
H	3.512375000000	7.544894000000	1.772426000000
H	2.929615000000	7.061902000000	0.165696000000

H	2.107441000000	6.471041000000	1.631272000000
C	7.668336000000	1.826953000000	-1.202556000000
C	6.521160000000	-0.292312000000	-0.883439000000
C	8.848876000000	1.299740000000	-0.688972000000
H	7.646606000000	2.862619000000	-1.533195000000
C	5.364174000000	-1.144310000000	-0.954790000000
C	7.731001000000	-0.841217000000	-0.353664000000
C	8.906258000000	-0.032945000000	-0.258069000000
H	9.737853000000	1.921427000000	-0.619219000000
C	5.407168000000	-2.440502000000	-0.538198000000
H	4.431725000000	-0.737854000000	-1.329649000000
C	7.773705000000	-2.200840000000	0.081519000000
C	-0.673344000000	0.218028000000	-3.061168000000
C	-2.211310000000	2.096739000000	-2.594015000000
C	-2.068739000000	2.629537000000	2.214863000000
C	-2.888910000000	3.270865000000	-0.026094000000
C	0.521565000000	-1.459684000000	-1.974092000000
C	-1.931142000000	0.839668000000	-3.286440000000
C	-0.572580000000	-1.200171000000	-2.883854000000
C	-3.554093000000	2.415202000000	-2.264957000000
C	0.421439000000	-2.501045000000	-1.056875000000
C	-3.018229000000	-0.021491000000	-3.596477000000
C	-1.722419000000	-2.035820000000	-2.927793000000
C	-3.888724000000	2.994631000000	-0.995507000000
C	-3.164529000000	2.970964000000	1.378161000000
C	-4.651891000000	1.564513000000	-2.672775000000
C	0.901620000000	-2.315373000000	0.285369000000
C	0.237652000000	-1.169199000000	2.794591000000
C	-0.874672000000	0.947077000000	3.294657000000
C	-0.024885000000	-2.993136000000	1.168309000000
C	-0.780844000000	-0.427740000000	3.484700000000
C	-0.350210000000	-2.431290000000	2.395170000000
C	-2.175988000000	1.566245000000	3.169505000000
C	-4.424576000000	2.407346000000	1.718167000000
C	-3.375557000000	0.817973000000	3.316581000000
C	-5.196374000000	2.506052000000	-0.612176000000
C	-0.782736000000	-3.309022000000	-1.010583000000

C	-1.056977000000	-3.614151000000	0.363613000000
C	-1.986856000000	-1.232408000000	3.536158000000
C	-1.719753000000	-2.471629000000	2.865898000000
C	10.108232000000	-0.612381000000	0.276119000000
C	6.607551000000	-3.019015000000	-0.006860000000
H	4.515373000000	-3.059870000000	-0.596057000000
C	8.983146000000	-2.749838000000	0.608443000000
C	10.145257000000	-1.908232000000	0.689581000000
H	10.994973000000	0.013221000000	0.343076000000
C	6.670895000000	-4.353353000000	0.427572000000
C	8.999166000000	-4.088790000000	1.029341000000
H	11.062428000000	-2.332009000000	1.091490000000
C	7.854980000000	-4.880288000000	0.938067000000
H	5.780754000000	-4.973982000000	0.360725000000
H	9.919808000000	-4.505854000000	1.430181000000
C	-2.915026000000	-1.440780000000	-3.419848000000
C	-4.387700000000	0.365934000000	-3.324975000000
C	-1.832764000000	-3.129335000000	-1.949786000000
C	-5.682039000000	1.624817000000	-1.655403000000
C	-4.528342000000	1.344708000000	2.674794000000
C	-5.458188000000	2.215199000000	0.721315000000
C	-3.276203000000	-0.638548000000	3.501920000000
C	-4.222351000000	-1.938343000000	-3.039636000000
C	-5.145194000000	-0.822746000000	-2.992340000000
C	-3.127501000000	-3.536390000000	-1.529029000000
C	-6.410095000000	0.480466000000	-1.325358000000
C	-2.391494000000	-3.744776000000	0.837569000000
C	-4.326889000000	-2.965249000000	-2.110626000000
C	-6.135989000000	-0.771432000000	-2.008937000000
C	-3.402893000000	-3.839168000000	-0.154478000000
C	-6.220276000000	1.033613000000	1.069418000000
C	-5.629426000000	0.490664000000	2.275243000000
C	-6.685512000000	0.177785000000	0.068259000000
C	-2.736127000000	-3.150505000000	2.138791000000
C	-4.331637000000	-1.458163000000	3.019220000000
C	-4.065362000000	-2.696818000000	2.347001000000
C	-5.531978000000	-0.884554000000	2.441950000000

C	-4.773983000000	-3.451675000000	0.119779000000
C	-5.099042000000	-2.890791000000	1.347358000000
H	7.886797000000	-5.914474000000	1.269724000000
C	-5.357363000000	-2.923499000000	-1.095277000000
C	-6.246379000000	-1.845739000000	-1.039080000000
C	-6.585642000000	-1.259619000000	0.243349000000
C	-6.021583000000	-1.777215000000	1.413368000000

}

[C60]B2

C	3.647033000000	5.011087000000	-0.392172000000
C	3.901928000000	6.133464000000	0.401403000000
C	4.662459000000	4.041288000000	-0.577995000000
C	4.477305000000	2.859074000000	-1.377544000000
C	5.473308000000	1.942744000000	-1.533771000000
H	3.524067000000	2.688597000000	-1.865949000000
C	5.931319000000	4.235165000000	0.054572000000
C	6.165423000000	5.393826000000	0.858530000000
C	6.973745000000	3.273395000000	-0.113600000000
C	5.133828000000	6.330083000000	1.015712000000
H	5.304029000000	7.213785000000	1.625342000000
C	7.450312000000	5.563159000000	1.479680000000
H	3.114401000000	6.870721000000	0.538700000000
C	6.754619000000	2.110716000000	-0.911628000000
H	5.303609000000	1.054784000000	-2.137840000000
C	8.241282000000	3.467848000000	0.516898000000
C	2.293340000000	4.867698000000	-1.030659000000
O	1.487266000000	3.902557000000	-0.279907000000
C	0.795790000000	2.983663000000	-0.977849000000
H	1.745704000000	5.812859000000	-0.987908000000
H	2.336735000000	4.536193000000	-2.068612000000

C	7.792050000000	1.175767000000	-1.062438000000
C	9.024425000000	1.374183000000	-0.444249000000
H	7.621674000000	0.290499000000	-1.670090000000
C	8.441190000000	4.645013000000	1.316348000000
H	7.614280000000	6.449043000000	2.088318000000
C	9.248664000000	2.507075000000	0.336822000000
H	9.408071000000	4.787448000000	1.792960000000
H	10.212761000000	2.655948000000	0.816977000000
H	9.816348000000	0.641170000000	-0.571137000000
O	0.826008000000	2.842632000000	-2.178280000000
C	-0.000147000000	2.132233000000	0.000355000000
C	-0.796171000000	2.983483000000	0.978636000000
C	-0.637735000000	0.844692000000	-0.481064000000
C	0.637545000000	0.844692000000	0.481632000000
O	-1.487469000000	3.902607000000	0.280822000000
O	-0.826610000000	2.842133000000	2.179024000000
C	-0.456784000000	0.206056000000	-1.821298000000
C	-1.871412000000	0.210639000000	0.086188000000
C	0.456655000000	0.205857000000	1.821780000000
C	1.871281000000	0.210828000000	-0.085705000000
C	-2.293815000000	4.867453000000	1.031669000000
C	0.771312000000	-0.239231000000	-2.277969000000
C	-1.642631000000	-0.552549000000	-2.126189000000
C	-2.508099000000	-0.551224000000	-0.961070000000
C	-1.954304000000	-0.234430000000	1.397378000000
C	-0.771399000000	-0.239614000000	2.278389000000
C	1.642572000000	-0.552680000000	2.126565000000
C	1.954215000000	-0.234054000000	-1.396956000000
C	2.508042000000	-0.551115000000	0.961447000000

C	-3.647350000000	5.010931000000	0.392867000000
H	-1.746249000000	5.812675000000	0.989367000000
H	-2.337454000000	4.535615000000	2.069506000000
C	0.849544000000	-1.416360000000	-3.113950000000
C	-1.584477000000	-1.679469000000	-2.952432000000
C	-3.281329000000	-1.677842000000	-0.666750000000
C	-2.737094000000	-1.413896000000	1.712693000000
C	-0.849517000000	-1.416865000000	3.114208000000
C	1.584528000000	-1.679719000000	2.952654000000
C	2.737120000000	-1.413398000000	-1.712435000000
C	3.281379000000	-1.677618000000	0.666975000000
C	-3.902096000000	6.133496000000	-0.400491000000
C	-4.662780000000	4.041039000000	0.578196000000
C	-0.308115000000	-2.121034000000	-3.460580000000
C	2.060237000000	-2.141155000000	-2.771912000000
C	-2.382093000000	-2.853574000000	-2.639547000000
C	-3.213207000000	-2.852694000000	-1.520533000000
C	-3.399266000000	-2.119387000000	0.702749000000
C	-2.060142000000	-2.141732000000	2.772072000000
C	0.308211000000	-2.121474000000	3.460744000000
C	2.382256000000	-2.853705000000	2.639611000000
C	3.399357000000	-2.118966000000	-0.702584000000
C	3.213372000000	-2.852594000000	1.520598000000
C	-5.133851000000	6.330216000000	-1.015056000000
H	-3.114567000000	6.870820000000	-0.537409000000
C	-4.477779000000	2.858638000000	1.377505000000
C	-5.931488000000	4.235014000000	-0.054648000000
C	-0.303186000000	-3.573689000000	-3.464194000000
C	2.069499000000	-3.535758000000	-2.784611000000

C	-1.587707000000	-4.027780000000	-2.956359000000
C	-3.287049000000	-4.026326000000	-0.668082000000
C	-3.402798000000	-3.572434000000	0.708233000000
C	-2.069275000000	-3.536337000000	2.784588000000
C	0.303419000000	-3.574131000000	3.464163000000
C	1.587983000000	-4.028031000000	2.956266000000
C	3.403029000000	-3.572011000000	-0.708265000000
C	3.287326000000	-4.026105000000	0.667987000000
C	-6.165426000000	5.393844000000	-0.858411000000
H	-5.303943000000	7.214068000000	-1.624499000000
C	-5.473784000000	1.942230000000	1.533260000000
H	-3.524661000000	2.688084000000	1.866112000000
C	-6.973884000000	3.273112000000	0.112949000000
C	0.861382000000	-4.267558000000	-3.132330000000
C	2.749744000000	-4.266617000000	-1.727319000000
C	-1.655428000000	-5.155906000000	-2.136646000000
C	-2.522739000000	-5.155414000000	-0.969423000000
C	-2.749451000000	-4.267114000000	1.727197000000
C	-0.861076000000	-4.268068000000	3.132207000000
C	1.655811000000	-5.156039000000	2.136399000000
C	2.523122000000	-5.155304000000	0.969174000000
C	-7.450153000000	5.563254000000	-1.479879000000
C	-6.754953000000	2.110310000000	0.910851000000
H	-5.304207000000	1.054139000000	2.137170000000
C	-8.241284000000	3.467686000000	-0.517788000000
C	0.790622000000	-5.442189000000	-2.283603000000
C	1.958387000000	-5.441993000000	-1.415349000000
C	-0.442561000000	-5.877705000000	-1.794202000000
C	-1.844986000000	-5.877289000000	0.093085000000

C	-1.957979000000	-5.442371000000	1.415067000000
C	-0.790195000000	-5.442574000000	2.283319000000
C	0.443013000000	-5.877904000000	1.793858000000
C	1.845437000000	-5.877100000000	-0.093432000000
C	-8.440796000000	4.644668000000	-1.317607000000
H	-7.614189000000	6.449561000000	-2.087883000000
C	-7.792385000000	1.175282000000	1.061175000000
C	-9.248636000000	2.506775000000	-0.338285000000
C	-0.559663000000	-6.325591000000	-0.416406000000
C	0.560157000000	-6.325592000000	0.415999000000
H	-9.407555000000	4.787169000000	-1.794446000000
C	-9.024618000000	1.373809000000	0.442740000000
H	-7.622123000000	0.289871000000	1.668652000000
H	-10.212630000000	2.655741000000	-0.818622000000
H	-9.816545000000	0.640733000000	0.569251000000

}

[C70]B2

C	-4.899644000000	4.330606000000	-0.518841000000
C	-6.003411000000	4.707060000000	0.252760000000
C	-3.842448000000	5.251438000000	-0.723124000000
C	-2.672844000000	4.941895000000	-1.503454000000
C	-1.675101000000	5.852628000000	-1.680363000000
H	-2.577667000000	3.962506000000	-1.959758000000
C	-3.932061000000	6.551606000000	-0.130640000000
C	-5.072609000000	6.909159000000	0.653550000000
C	-2.883425000000	7.502930000000	-0.319445000000
C	-6.097780000000	5.968972000000	0.828203000000
H	-6.969174000000	6.234045000000	1.421439000000

C	-5.136721000000	8.222405000000	1.233622000000
H	-6.808692000000	3.991746000000	0.402563000000
C	-1.738396000000	7.162521000000	-1.100134000000
H	-0.802194000000	5.591847000000	-2.273956000000
C	-2.972975000000	8.800749000000	0.272024000000
C	-4.874205000000	2.946840000000	-1.108492000000
O	-4.036761000000	2.062212000000	-0.292365000000
C	-3.046914000000	1.387486000000	-0.899317000000
H	-5.871211000000	2.500937000000	-1.083468000000
H	-4.498533000000	2.916494000000	-2.131278000000
C	-0.717919000000	8.112048000000	-1.273567000000
C	-0.815543000000	9.375539000000	-0.695382000000
H	0.153985000000	7.848598000000	-1.867205000000
C	-4.136143000000	9.126041000000	1.050226000000
H	-6.010322000000	8.479833000000	1.827457000000
C	-1.928827000000	9.717150000000	0.071155000000
H	-4.199284000000	10.115908000000	1.495720000000
H	-1.998258000000	10.704230000000	0.521746000000
H	-0.017271000000	10.098443000000	-0.839289000000
C	-2.310742000000	0.542198000000	0.122001000000
O	-2.756761000000	1.469812000000	-2.072063000000
C	-3.182208000000	0.153298000000	1.311680000000
C	-0.852109000000	0.873110000000	0.391945000000
C	-1.295903000000	-0.479246000000	-0.342248000000
O	-4.126115000000	-0.710295000000	0.900072000000
O	-3.047911000000	0.547936000000	2.445095000000
C	-0.162608000000	0.722816000000	1.702042000000
C	0.035915000000	1.778604000000	-0.393086000000
C	-0.954552000000	-1.737565000000	0.390981000000

C	-0.747373000000	-0.681350000000	-1.717966000000
C	-5.048029000000	-1.171757000000	1.934677000000
C	-0.023777000000	-0.492534000000	2.337262000000
C	0.904697000000	1.681602000000	1.773186000000
C	0.377366000000	1.536012000000	-1.709246000000
C	1.029251000000	2.322972000000	0.497492000000
C	-0.481722000000	-2.707421000000	-0.563707000000
C	-0.425196000000	-1.737220000000	1.675481000000
C	-0.352920000000	-2.060119000000	-1.851497000000
C	-0.014980000000	0.293091000000	-2.377670000000
C	-6.062821000000	-2.089225000000	1.312819000000
H	-5.525087000000	-0.296555000000	2.382369000000
H	-4.454799000000	-1.661068000000	2.713360000000
C	1.162953000000	-0.761690000000	3.119668000000
C	2.032522000000	1.498231000000	2.621184000000
C	1.694258000000	1.905113000000	-2.190701000000
C	2.289654000000	2.796168000000	0.036542000000
C	0.433951000000	-3.699139000000	-0.187334000000
C	0.544190000000	-2.741045000000	2.060190000000
C	0.688268000000	-2.431380000000	-2.712991000000
C	1.078864000000	-0.079237000000	-3.242506000000
C	-7.394287000000	-1.670678000000	1.237738000000
C	-5.706288000000	-3.372092000000	0.830893000000
C	2.164744000000	0.224506000000	3.327006000000
C	1.509264000000	-2.142248000000	2.955950000000
C	3.205797000000	2.216840000000	2.275117000000
C	2.634597000000	2.581257000000	-1.368191000000
C	2.121829000000	0.917386000000	-3.136854000000
C	3.332966000000	2.857586000000	0.997457000000

C	0.958384000000	-3.709188000000	1.151028000000
C	1.519731000000	-4.062979000000	-1.074271000000
C	1.418245000000	-1.416650000000	-3.420266000000
C	1.644147000000	-3.441804000000	-2.309310000000
C	-8.388116000000	-2.486688000000	0.706827000000
H	-7.657522000000	-0.683453000000	1.609981000000
C	-4.356642000000	-3.869336000000	0.864298000000
C	-6.722610000000	-4.216843000000	0.283988000000
C	3.468840000000	-0.250013000000	3.632807000000
C	2.863010000000	-2.575940000000	2.995066000000
C	4.517603000000	1.756683000000	2.678389000000
C	4.005204000000	2.435752000000	-1.716743000000
C	3.494192000000	0.580050000000	-3.290468000000
C	4.724894000000	2.797695000000	0.604240000000
C	2.354497000000	-4.100764000000	1.098917000000
C	2.700246000000	-4.320884000000	-0.275392000000
C	2.815010000000	-1.804291000000	-3.477910000000
C	2.953998000000	-3.057264000000	-2.794356000000
C	-8.077781000000	-3.765526000000	0.223856000000
H	-9.416030000000	-2.135651000000	0.664329000000
C	-4.042974000000	-5.110217000000	0.398211000000
H	-3.568613000000	-3.232726000000	1.250425000000
C	-6.391119000000	-5.517771000000	-0.203375000000
C	3.813356000000	-1.632271000000	3.469354000000
C	4.646355000000	0.543114000000	3.343477000000
C	3.302609000000	-3.591198000000	2.025313000000
C	5.469635000000	2.124512000000	1.649682000000
C	4.429111000000	1.448098000000	-2.665504000000
C	5.054492000000	2.587830000000	-0.729412000000

C	3.854056000000	-0.836568000000	-3.462441000000
C	4.005179000000	-4.033071000000	-0.762459000000
C	4.137037000000	-3.375772000000	-2.072298000000
C	-9.078094000000	-4.637156000000	-0.328837000000
C	-5.042206000000	-5.981124000000	-0.150375000000
H	-3.013216000000	-5.457983000000	0.428826000000
C	-7.407090000000	-6.362755000000	-0.747030000000
C	5.207842000000	-1.700426000000	3.079451000000
C	5.735125000000	-0.352842000000	3.012924000000
C	4.656570000000	-3.577785000000	1.594309000000
C	6.516589000000	1.261503000000	1.321635000000
C	5.745064000000	0.985848000000	-2.270435000000
C	6.145340000000	1.700072000000	-1.075620000000
C	5.116430000000	-1.279444000000	-2.983873000000
C	5.002618000000	-3.794963000000	0.219569000000
C	5.256031000000	-2.531485000000	-2.298398000000
C	-8.758592000000	-5.876140000000	-0.791831000000
H	-10.104904000000	-4.281791000000	-0.368109000000
C	-4.737554000000	-7.263660000000	-0.635659000000
C	-7.055491000000	-7.636899000000	-1.219392000000
C	5.621426000000	-2.653723000000	2.158265000000
C	6.652871000000	-0.005488000000	2.018702000000
C	6.862510000000	1.044199000000	-0.072193000000
C	6.081454000000	-0.352689000000	-2.424433000000
C	6.181668000000	-3.001357000000	-0.072496000000
C	6.305756000000	-2.380923000000	-1.308149000000
H	-9.526917000000	-6.523821000000	-1.207156000000
C	-5.734943000000	-8.080647000000	-1.162957000000
H	-3.708877000000	-7.613331000000	-0.596224000000

H	-7.829096000000	-8.279321000000	-1.632940000000
C	6.579634000000	-2.303405000000	1.131741000000
C	7.086420000000	-1.002392000000	1.056873000000
C	7.215741000000	-0.354302000000	-0.234008000000
C	6.833396000000	-1.035657000000	-1.393571000000
H	-5.482187000000	-9.070054000000	-1.534080000000

}
